# Transformation and other factors of the peptide mass spectrometry pairwise peak-list comparison process

**DOI:** 10.1186/1471-2105-6-285

**Published:** 2005-11-30

**Authors:** Witold E Wolski, Maciej Lalowski, Peter Martus, Ralf Herwig, Patrick Giavalisco, Johan Gobom, Albert Sickmann, Hans Lehrach, Knut Reinert

**Affiliations:** 1 Max Planck Institute for Molecular Genetics, Ihnestraβe 63-73, D-14195 Berlin, Germany; 2 Institute for Computer Science, Free University Berlin, Takustr. 9, D-14195 Berlin, Germany; 3 DFG Research Center for Experimental Biomedicine, University of Würzburg, Versbacherstr. 9, D-97078 Würzburg, Germany; 4 Max Delbrück Center for Molecular Medicine, Robert-Roessle-Str. 10, D-13125 Berlin-Buch, Germany; 5 Boyce Thompson Institute for Plant Research, Tower Road, Ithaca 14850, NY, USA; 6 Institute for Medical Informatics, Biometry and Epidemiology; Charite University Medicine Berlin, Hindenburgdamm 30 (HBD 30), 12200 Berlin; 7 School of Mathematics and Statistics, Merz Court, University of Newcastle upon Tyne, NE1 7RU, UK

## Abstract

**Background:**

Biological Mass Spectrometry is used to analyse peptides and proteins. A mass spectrum generates a list of measured mass to charge ratios and intensities of ionised peptides, which is called a peak-list. In order to classify the underlying amino acid sequence, the acquired spectra are usually compared with synthetic ones. Development of suitable methods of direct peak-list comparison may be advantageous for many applications.

**Results:**

The pairwise peak-list comparison is a multistage process composed of matching of peaks embedded in two peak-lists, normalisation, scaling of peak intensities and dissimilarity measures. In our analysis, we focused on binary and intensity based measures. We have modified the measures in order to comprise the mass spectrometry specific properties of mass measurement accuracy and non-matching peaks. We compared the labelling of peak-list pairs, obtained using different factors of the pairwise peak-list comparison, as being the same or different to those determined by sequence database searches. In order to elucidate how these factors influence the peak-list comparison we adopted an analysis of variance type method with the partial area under the ROC curve as a dependent variable.

**Conclusion:**

The analysis of variance provides insight into the relevance of various factors influencing the outcome of the pairwise peak-list comparison. For large MS/MS and PMF data sets the outcome of ANOVA analysis was consistent, providing a strong indication that the results presented here might be valid for many various types of peptide mass measurements.

## Background

In recent years, mass spectrometry (MS) has emerged as a powerful technique to identify proteins in biological samples [1-4]. For their identification, proteins are usually cleaved into peptides by a protease of known and restricted cleavage specificity, *e.g. *trypsin. The resulting cleavage products can then be analysed by Peptide Mass Fingerprinting (PMF) [5] or subjected to MS/MS fragment ion analysis [6,7]. A PMF is a highly specific set of peptide molecular masses derived from one isolated protein. PMFs are employed to identify the analysed protein in large protein sequence databases by matching the determined peptide molecular masses to values calculated from the amino acid sequences in the database. Similarly, MS/MS spectra serve for protein identification by comparing the determined peptide fragment ion masses against predicted ones from amino acid sequence data and fragmentation characteristics of the employed MS instrumentation [8].

Before performing database searches, the MS spectra are processed and the most informative features, namely the monoisotopic peaks are extracted. The procedure consists of several steps and includes smoothing, baseline subtraction, peak-extraction and monoisotopic peak determination [9,10]. A spectrum pre-processing usually requires proprietary software provided by the instrument vendors. It generates a list of mass over charge (*m/z*) values of the monoisotopic peaks and either the area under or the height of those peaks are obtained. The set of *m/z *and intensity value pairs is called a *peak-list*. In case of PMF datasets, the peak-lists have on average 36 peak/intensity pairs compared to *e.g. *100,000 data points of the unprocessed spectra.

The sensitivity and specificity of the peptide identification using database searches might be increased by several methods. These usually include calibration [11-13], identification of non-peptide peaks [12,14,15], identification and removal of low-quality spectra [16,17] or validation of the search results using machine-learning algorithms [18,19].

### The subtractive analysis technique

The sensitivity and specificity of peptide and protein identification can further be increased by the pairwise comparison of the peak-lists [11,20-22]. Yates et al. [20] applied, as a measure of peak-list similarity, the cross-correlation score normalised by the auto-correlation of the spectra. They demonstrated that when using this measure, the MS spectra could be correctly classified according to their peptide content even if acquired on two different instruments, namely a Triple-Quadrupole Tandem (TSQ) or Quadrupole Ion Trap (LCQ) mass spectrometers.

They suggested, as part of a "subtractive analysis technique", using pairwise spectra comparisons to search MS spectra against a library of identified spectra before database searching. Tandem mass spectra (unique to an experiment) could be targeted for database searches or *de novo *interpretation.

A significant portion of identical peptides is analysed and identified many times even when the instrument control software attempts to prevent the repeated isolation and fragmentation of particular peptides in order to increase the diversity of acquired spectra. Gentzel et al. [11] used the cross-correlation measure for MS/MS spectra comparison. They computed the similarity score for two parts of the spectra. If these parts exhibited a satisfying similarity score, the spectra were assumed to be identical. Tabb et al. [22] explored the performance of the normalised dot-product (spectral angle) algorithm to identify duplicated samples. The advantage of the dot-product measure over the cross-correlation algorithm lies in its computational speed. Based on this measure, Tabb et al. [22] and Beer et al. [21] developed software to identify the duplicated MS/MS spectra. The analysis time saved by subtractive analysis can be used instead to perform more extensive searches in other databases, *i.e. *expressed sequence tag (EST) databases, or to apply computationally demanding, mutation-tolerant search algorithms [23], which depend on partial spectra interpretation [24-26].

Pairwise spectra comparison can also be put to use "as an informative marker to identify organisms or some other feature of an organism" [20]. For example, Svetnik and Liaw [27] used pairwise spectra comparison to detect novel outliers in large-scale cosmid screening experiments [28]. They used the Pearson and Spearman correlation measures, as well as the Euclidean distance to compute the distances of the spectra, followed by sequential clustering. Serum protein and peptide fingerprints were used in diagnostic medicine to distinguish healthy individuals from those with cancer [29-32].

### The pairwise peak-list comparison process

Previously, only the performance of the dot-product measure has been compared to the similarity index using MS/MS spectra of structural isomers [33]. Therefore, in our work we have reviewed a large group of dissimilarity measures and examined how these can be extended to include the mass spectrometry specific property of mass measurement accuracy. A new parameter weight of non-matching peaks (*θ*) was introduced into the computation of distance measures. We have studied the Euclidean and the Manhattan distance, the covariance, the sum of agreeing intensities and the spectral angle. We have also examined the impact of the intensity scaling on the outcome of intensity based measures [34,35]. In addition, we have performed a systematic study of various intensity transformations [22] in order to determine the best variance stabilising transformation. Furthermore, we investigated quantitative measures, *i.e. *Huberts Γ or the relative mutual information measure [36]. The combination of these factors resulted in 96 choices of the comparison process for the binary measures and 2688 approaches for the intensity based measures. The first aim of the work presented here was to determine the pairwise peak-list comparison approach with highest sensitivity and specificity for the grouping of spectra. The second goal was to determine which factors studied had the highest effect on the outcome of the clustering, in order to foster the understanding of the pairwise peak-list comparison process. While the first goal could be easily achieved by ranking the various peak-list comparison approaches, the second goal was approached by analysis of variance (ANOVA) techniques. The partial area under the receiver operator characteristic (ROC) curve, determined for high sensitivity and specificity values was used as the dependent variable, while the various choices for the comparison process were the factors in the ANOVA.

### Evaluation framework

**Figure 1:**
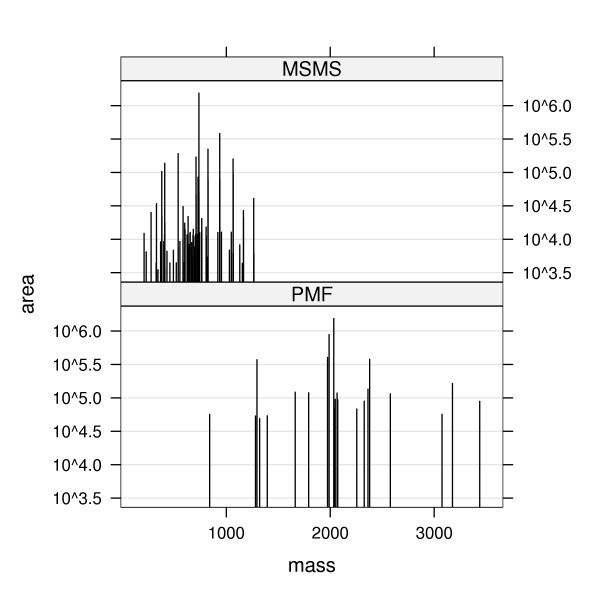
Example of a peak-list stick spectrum for fragment ion MS/MS (top panel) and PMF(bottom panel). X-axis – mass of the peaks, Y-axis – area under the peak.

PMF and MS/MS data represent mass spectrometric peptide peak-lists. While the PMF measurement is characterised by high-mass resolution, large mass range and the production of relatively few peaks, the MS/MS spectra have a lower-mass resolution, a smaller mass range and a higher number of peaks. Figure 1 presents examples of peak-lists for fragment ion MS/MS and PMF, respectively. We have analysed the pairwise comparison process using both datasets in order to determine if the differences of the data require different configuration of the pairwise comparison.

In order to determine the sensitivity and specificity of the measures used for classification, the grouping induced by the measures must be compared with the true cluster membership of the spectra. However, we did not have a dataset with a large number of groups of spectra and with identities known a-priori.

Therefore, in our study we used PMF and MS/MS spectra resulting from studies of different proteomes. The identities of the spectra were determined by database searches (cf. Methods). We assumed that the database searches resulted in *true *identification of the peptides and proteins.

In the PMF dataset, only 176 proteins out of 668 were identified by a single spectrum, while the remaining 492 protein identifications resulted from 2160 database searches. The amount of duplicated samples in the two datasets (Table 1) was significant and recognising them could for example, significantly reduce the number of searches necessary to identify all proteins.

**Table 1:** Number of clusters of given cluster size *N*. The columns 2 and 3 describe the cluster size in the PMF- and the MS/MS datasets. Number of spectra – number of peak-lists submitted for database search, identified spectra – spectra assigned to a database ID with an either significant probability based Mowse score (PMF-data) or to a peptide sequence with *Xcorr *> 2, and an ion coverage > 20% (MS/MS-data) given a parent peptide charge *z *= 2. Identified proteins/peptides – the number of uniquely identified proteins or peptides. ^*A *^– approximate number of spectra derived from ion fragments of peptides with charge *z *= 2. ^*B *^– The number of spectra with charge *z *= 2 of the parent ion (≈ 53% of all identified spectra).

	Dataset	
	PMF	MS/MS

number of spectra	4532	≈ 200000^*A*^
number of identified spectra	2336	26507^*B*^
*N *= 1	176	5718
*N *∈ (1,5]	392	1965
*N *∈ (5, 10]	66	388
*N *∈ (10, 25]	31	354
*N *∈ (25, 50]	2	111
*N *> 50	1	43
Identified: proteins (PMF)|peptides (MS/MS)	668	8579

According to the protein database identifier (ID), in case of the PMF data or the peptide sequence and the same parent ion charge *z *= 2 for MS/MS data, we defined a peak-lists pair (*X*, *Y*) to be *within *a cluster if it was assigned to the same protein ID or peptide sequence. Similarly, two peak-lists were defined to lie *between *the clusters if their database IDs differed. This assignment of the peak-lists pairs as *within *and *between *was recognised as the *true condition status*. The assignment induced by the pairwise comparison approach for a given thresholds of the discriminatory variable was compared with this *true condition status *and the sensitivities and specificities were computed.

The spectra in the large group of unidentified peak-lists could not be used in this study. This is because we could not infer that all these spectra were derived from the same peptide/protein. Secondly, we could not assume that an un-identified spectrum was not obtained in a measurement of the same peptide as any of the spectra assigned to a database identifier or peptide sequence. The identification of a protein/peptide often fails because of a signal to noise ratio that is too small. If we would treat this group likewise the clusters formed by identified spectra we would then introduce an error during the determination of false positive/false negative rates.

Using the data, where the identities of samples were determined by database search algorithms we were able to examine whether the pairwise peak-list comparison made equal or different assignments to a group, in relation to the database search algorithm. We were not able to disclose if any of these measures had higher sensitivities and specificities than the database search algorithms used. However, these data were sufficient to expose relative differences between pairwise peak-list comparison approaches, as well as the degree by which the various factors of the comparison process influenced the outcome.

The computation of the pairwise peak-list distances was performed using the in-house developed R [37] package msbase, which is available from the BioConductor Project [38] web page [39].

## Results and Discussion

### The factors of the pairwise peak-list comparison

Table 2 summarises the factors, which can influence the outcome of a pairwise peak-list comparison. The first step in the comparison of the peak-lists is to determine matching and non-matching peaks with given mass measurement accuracy. If one peak is ambiguously assigned to several peaks in the second peak-list (cf. Methods – Figure 6), the non-crossing matching can be computed. The next element to be considered is whether the mass measurement accuracy should be modelled [45,46] using Equation 2. Modelling of the mass differences between matching masses did not affect the non-matching peaks. The influence of non-matching peaks on the pairwise peak-list comparison was modulated by increasing or decreasing their weight by two-fold (using the parameter *θ *in the dissimilarity equations (cf. Methods)).

**Table 2:** Factors considered in the comparison process and their levels. Column 1 – Factors: identification of factors, Column 2 – Levels: short summary of the levels (For more details please refer to the Methods section). Column 3 – Number: number of levels. Int. – comparisons considering the intensities; Bin. – binary measures.

	**Factors**	**Levels**				**Number**	
						Int.	Bin

1	non crossing matching	yes		no		2	
2	weighting match accuracy	yes		no		2	
3	weight of non-matching peaks	0.5	1		2	3	
4	intensity transformation	I	I MathType@MTEF@5@5@+=feaafiart1ev1aaatCvAUfKttLearuWrP9MDH5MBPbIqV92AaeXatLxBI9gBaebbnrfifHhDYfgasaacH8akY=wiFfYdH8Gipec8Eeeu0xXdbba9frFj0=OqFfea0dXdd9vqai=hGuQ8kuc9pgc9s8qqaq=dirpe0xb9q8qiLsFr0=vr0=vr0dc8meaabaqaciGacaGaaeqabaqabeGadaaakeaadaGcaaqaaiabdMeajbWcbeaaaaa@2DE4@	log(*I*)	rank(I)	4	0
5	intensity normalisation	tic(I)	||*I*||	*S*(*I*)	Z(I)	4	0
6	alignment length	M=M1X+M1Y−M11XY MathType@MTEF@5@5@+=feaafiart1ev1aaatCvAUfKttLearuWrP9MDH5MBPbIqV92AaeXatLxBI9gBaebbnrfifHhDYfgasaacH8akY=wiFfYdH8Gipec8Eeeu0xXdbba9frFj0=OqFfea0dXdd9vqai=hGuQ8kuc9pgc9s8qqaq=dirpe0xb9q8qiLsFr0=vr0=vr0dc8meaabaqaciGacaGaaeqabaqabeGadaaakeaacqWGnbqtcqGH9aqpcqWGnbqtdaqhaaWcbaGaeGymaedabaGaemiwaGfaaOGaey4kaSIaemyta00aa0baaSqaaiabigdaXaqaaiabdMfazbaakiabgkHiTiabd2eannaaDaaaleaacqaIXaqmcqaIXaqmaeaacqWGybawcqWGzbqwaaaaaa@3D52@		*M *= *const*		2	
7	distance measure	See Methods Section				7	4
Product of levels for nonzero factors:						2688	96

The length of the aligned peak-lists either equals the sum of the peaks in both peak-lists minus the number of peaks matching or is user-defined. In Equation 3, we set *N *= 250 for the PMF dataset and *N *= 400 for the MS/MS dataset, which in both cases was approximately twice the length of the longest peak-list. In case of the intensity based measures the *missing *peak pairs were augmented by peaks of zero intensity. Further elements which affected only the intensity-based measures included the transformation and scaling of peak intensities. The distance measures (cf. Methods) were the last of the examined factors. Section 'Features of the pairwise peak-list comparison and their properties' in the Appendix section provides a descriptive analysis of the features of the pairwise peak-list comparison in order to introduce the data and to motivate the use of various factors of the peak-list comparison approach.

The intensity-based measures contained 2688 sets of factors while the binary measures covered 96 sets. To determine which of these factors were important and how they influenced the scores, we applied analysis of variance techniques (ANOVA). We first performed the ANOVA on the PMF dataset. Afterwards, we examined if the obtained linear model could be used to explain the properties of the pairwise peak-list comparison process computed on the MS/MS dataset.

### The evaluation scores

**Figure 2:**
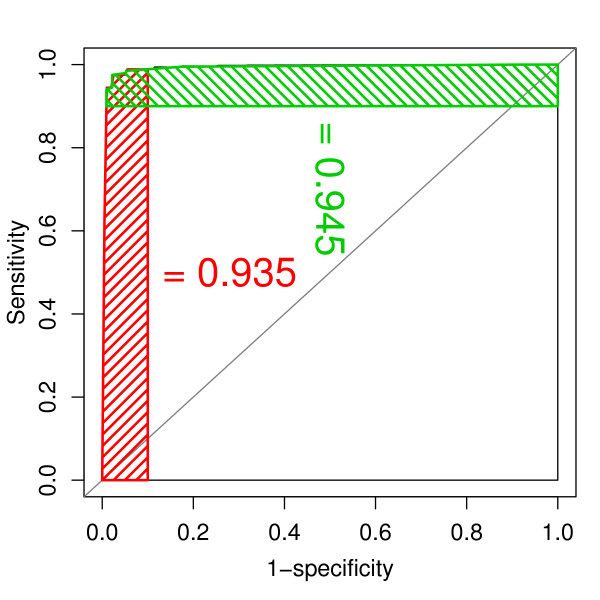
Receiver Operator Characteristic curve – The sensitivity (TP-rate) is plotted against *FP *= 1 - *specificity *using the number of matching peaks as the discriminatory variable. Red dashed area: sensitivity-PAUC – partial area under the ROC curve for FP-rate ∈ [0, 0.1]. Green dashed area: specificity-PAUC – partial area under the ROC curve for sensitivities ∈ [0.9, 1].

In order to evaluate the capability of pairwise comparison approaches to identify peak-list pairs as being *within *or *between *cluster we used the partial area of interest under the *Receiver Operating Characteristic *(ROC) curve (PAUC) [44]. The ROC curve was generated by drawing the sensitivity=TPTP+FN
 MathType@MTEF@5@5@+=feaafiart1ev1aaatCvAUfKttLearuWrP9MDH5MBPbIqV92AaeXatLxBI9gBaebbnrfifHhDYfgasaacH8akY=wiFfYdH8Gipec8Eeeu0xXdbba9frFj0=OqFfea0dXdd9vqai=hGuQ8kuc9pgc9s8qqaq=dirpe0xb9q8qiLsFr0=vr0=vr0dc8meaabaqaciGacaGaaeqabaqabeGadaaakeaacqWGZbWCcqWGLbqzcqWGUbGBcqWGZbWCcqWGPbqAcqWG0baDcqWGPbqAcqWG2bGDcqWGPbqAcqWG0baDcqWG5bqEcqGH9aqpdaWcaaqaaiabdsfaujabdcfaqbqaaiabdsfaujabdcfaqjabgUcaRiabdAeagjabd6eaobaaaaa@450D@, where *TP *– are true positives, *FN *– false negatives, against the 1−specificity=FPrate=FPFP+TN
 MathType@MTEF@5@5@+=feaafiart1ev1aaatCvAUfKttLearuWrP9MDH5MBPbIqV92AaeXatLxBI9gBaebbnrfifHhDYfgasaacH8akY=wiFfYdH8Gipec8Eeeu0xXdbba9frFj0=OqFfea0dXdd9vqai=hGuQ8kuc9pgc9s8qqaq=dirpe0xb9q8qiLsFr0=vr0=vr0dc8meaabaqaciGacaGaaeqabaqabeGadaaakeaacqaIXaqmcqGHsislcqWGZbWCcqWGWbaCcqWGLbqzcqWGJbWycqWGPbqAcqWGMbGzcqWGPbqAcqWGJbWycqWGPbqAcqWG0baDcqWG5bqEcqGH9aqpcqWGgbGrcqWGqbaudaWgaaWcbaGaemOCaiNaemyyaeMaemiDaqNaemyzaugabeaakiabg2da9maalaaabaGaemOrayKaemiuaafabaGaemOrayKaemiuaaLaey4kaSIaemivaqLaemOta4eaaaaa@4F66@, where *FP *– false positives, *TN *– true negatives, for the same value of the discriminatory variable, *i.e. *the number of matching peaks as shown in Figure 2. For 4 matches we determined a specificity of 99% and sensitivity of 95%.

We were particularly interested in the sensitivity of the pairwise peak-list comparison only for small values of the *FP*-rate. Therefore, we computed the *Partial Area *under the ROC curve (PAUC) for 1 - *specificity *∈ [0, 0.1] (red-dashed region Figure 2), denoted by sensitivity-PAUC. Moreover, we were also interested in the specificities when high sensitivities are required (*sensitivity *∈ [0.9, 1]), further abbreviated specificity-PAUC. Hence, we computed the PAUC for the area indicated in Figure (2) by the green-dashed region. Both sensitivity-PAUC and specificity-PAUC were utilised as the dependent variable in the analysis of variance.

### ANOVA of the pairwise peak-list comparison approaches

The aim of the statistical analysis was to evaluate different strategies of the pairwise peak-list comparison with respect to the sensitivity and specificity partial area under the ROC curve (PAUC). A possible strategy to decide which pairwise comparison approach performs best would have been to choose the one with the largest partial area under the curve. However, we were also interested in determining the influence of and the dependence between the factors of the pairwise peak-list comparison on the outcome of the classification.

Each strategy of the pairwise peak-list comparison was defined by the combination of seven factors as given in Table 2. The whole set of pairwise peak-list comparison strategies shows a completely balanced factorial structure analogous to those used in analysis of variance (ANOVA) [42] with PAUCs as dependent variables and the specific strategies of pairwise peak-list comparison as combinations of factor levels. However, due to multi-modality our data could not be transformed to approximate normality. Thus, we could not calculate *F*-ratios and related statistical tests of significance. To assess the significance of the factors, we therefore used the relative sum of squares (%SSQ) and the relative mean sum of squares (%MSQ), defined as the ratio of the SSQ or MSQ with respect to the total SSQ or MSQ. We did not calculate *F*-ratios or P-values for factors and interactions, due to the mentioned deviation from normality. A large value of the relative sums of squares (%SSQ) for a factor in Tables 3 and 4 indicates its importance for the correct classification of peak-lists.

**Table 3:** Influence of factors specifying the pairwise peak-list comparison on partial areas under the ROC curve for binary PMF and MS/MS data. For each of the 96 pairwise comparison approaches, sensitivity-PAUC (sensitivity given FP-rate ∈ [0, 0.1]) and specificity-PAUC (specificity given sensitivity ∈ [0.9, 1]) (Figure 2) were determined. A partitioning of sums of squares was performed analogously to analysis of variance. Column names: Factors – identification of factors; df – degrees of freedom (DF, number of factor levels – 1); %SSQ – relative sum of squares (%SSQ = *SSQ*/∑*SSQ*); %MSQ – relative mean sum of squares (%MSQ = *MSQ*/∑*MSQ*), where *MSQ *= *SSQ*/*DF*. %MSQ measures the importance of a specific factor for the size of specificity-PAUC and sensitivity-PAUC. × denotes interactions between factors. measure – distance measure, noncross – non crossing matching, length – alignment length, *θ *– weight of non-matching peaks, residual – unexplained %SSQ or %MSQ, total – column sum of %SSQ, df, %MSQ.

		PMF				MS/MS			
		specificity – PAUC		sensitivity – PAUC		specificity – PAUC		sensitivity – PAUC	

	Model with main effects								

Factors	df	%SSQ	%MSQ	%SSQ	%MSQ	%SSQ	%MSQ	%SSQ	%MSQ

measure	3	10	14.9	10.9	16.1	4.4	8.5	2.7	5.5
*θ*	2	17.3	38.1	17.0	37.7	14.3	42.0	15.9	48.2
length	1	10	43.9	9.7	43.0	6.4	37.3	5.8	35.4
weight	1	0	0	0	0	0.0	0.2	0.4	2.2
noncross	1	0	0	0	0	1.2	7.1	0.6	3.5
residual	87	62.5	3.2	62.4	3.2	73.7	5.0	74.6	5.2

total	95	100	100	100	100	100	100	100	100

	Final model N								

Factors	df	%SSQ	%MSQ	%SSQ	%MSQ	%SSQ	%MSQ	%SSQ	%MSQ

measure	3	10.2	8.5	10.9	9.2	4.4	4.8	2.7	3.0
*θ*	2	17.3	21.7	17.0	21.5	14.3	23.6	15.9	26.0
length	1	10.0	25.0	9.7	24.5	6.4	21.0	5.8	19.1
measure × *θ*	6	7.5	7.3	17.7	7.4	16.3	9.0	17.2	9.4
measure × length	3	10.1	8.4	10.0	8.4	7.3	8.0	6.5	7.1
*θ *× length	2	17.3	21.7	17.0	21.5	14.4	23.8	15.6	25.6
measure × *θ *× length	6	17.5	7.3	17.7	7.4	416.0	8.8	16.4	8.9
residual	72	0	0	0	0	20.9	1.0	19.9	0.9

total	95	100	100	100	100	100	100	100	100

**Table 4:** Influence of factors specifying the pairwise peak-list comparison on partial areas under the ROC curve for intensity PMF and MS/MS data. For each of the 2688 pairwise peak-list comparison approaches, sensitivity-PAUC (sensitivity given FP-rate ∈ [0, 0.1]) and specificity-PAUC (specificity given sensitivity ∈ [0.9, 1]) (Figure 2) were determined. A partitioning of sums of squares was performed analogously to analysis of variance. Column names: Factors – identification of factors; df – degrees of freedom (DF, number of factor levels - 1); %SSQ – relative sum of squares (%SSQ = *SSQ*/∑*SSQ*); %MSQ – relative mean sum of squares (%MSQ = *MSQ*/∑*MSQ*), where %MSQ = *MSQ*/∑*MSQ*. %MSQ measures the importance of a specific factor for the size of sensitivity-PAUC and specificity-PAUC. × denotes interactions between factors. measure – distance measure, noncross – non crossing matching, length – alignment length, *θ *– weight of non-matching peaks, trans – peak intensity transformation, residual – unexplained %SSQ or %MSQ, total – column sum of %SSQ, df, %MSQ.

		PMF				MS/MS			
		specificity – PAUC		sensitivity – PAUC		specificity – PAUC		sensitivity – PAUC	

	Model with main effects								

Factors	df	%SSQ	%MSQ	%SSQ	%MSQ	%SSQ	%MSQ	%SSQ	%MSQ

measure	6	25.2	36.1	20	29	14.9	20.9	15	21.2
scale	3	15.7	45.1	22.3	64.6	23.9	66.8	25.1	71.1
*θ*	2	3.1	13.2	0.7	2.8	1.4	5.7	0.9	3.9
length	1	0.5	4.1	0.4	3.2	0.3	2.4	0.1	1.1
weight	1	0	0.1	0	0	0.2	2.0	0.2	1.7
noncross	1	0	0	0	0	0	0.3	0.0	0.1
trans	3	0.4	1.3	0.1	0.2	0.6	1.7	0.3	0.8
residual	2670	55.1	0.2	56.6	0.2	58.6	0.2	58.4	0.2

total	2687	100	100	100	100	100	100	100	100

	Final model								

Factors	df	%SSQ	%MSQ	%SSQ	%MSQ	%SSQ	%MSQ	%SSQ	%MSQ

measure	6	25.2	29.7	20	23	14.9	17.4	15	17.4
scale	3	15.7	37.1	22.3	51.2	23.9	55.8	25.1	58.1
*θ*	2	3.1	10.8	0.7	2.2	1.4	4.7	0.9	3.2
length	1	0.5	3.3	0.4	2.5	0.3	2.0	0.1	0.9
measure × scale	18	33.4	13.2	41.2	15.8	43.4	16.9	44	17
measure × *θ*	12	6.3	3.7	1.9	1.1	3.7	2.2	2.3	1.3
measure × length	6	1.8	2.1	3.6	4.1	0.9	1.1	1.9	2.2
residual	2639	14	0	10	0	11.4	0	10.7	0

total	2687	100	100	100	100	100	100	100	100

### The ANOVA results

The high value of the %SSQ or %MSQ value in Table 3 and 4 reflect the change (variance) of the response variable PAUC caused by a factor or combination of factors. In case of the intensity based measures, the high value of the relative mean sum of squares (%MSQ) (Table 4, top panel) for the factor 'scale' (intensity scaling procedure) and 'measure' (dissimilarity measure) indicates that these factors were crucial for the correct classification of peak-lists. The small values of the %MSQ for the factors 'weight of match accuracy' (weight) and 'computing the non-crossing matching' (noncross) shows that these factors had a negligible impact on the result of pairwise peak-list comparison.

A large value of %MSQ or %SSQ of an interaction term (denoted by × in Table 3 and 4, bottom panel) demonstrates that some combinations of factors were more useful than others. The high value of %SSQ for the interaction *measure *× *scale *reflects that, for example, the measure sum of agreeing intensities performed better in combination with the vector length scaling (N) or root-mean-square scaling (S), than with the total ion count scaling (T) or with the *z*-score scaling (Z). We concluded that the crucial factors of the pairwise peak-list comparison were the measure and peak intensity scaling, followed by the weight of non-matching peaks and the length of the peak-list.

In case of binary measures, the high %MSQ value (Table 3, column %MSQ) of factors 'measure', 'weight of non-matching peaks' *θ*, 'peak-list length' *N *as well as of their interactions indicates that their are crucial for the outcome of the pairwise comparison.

In order to examine the extend to which the properties of the pairwise peak-list comparison, determined for the PMF data set can be generalised to other types of mass spectrometric data, we applied the ANOVA to the MS/MS dataset (Tables 3 and 4, right panel). The main difference between these two datasets is that the computation of the non-crossing matching which influenced the PAUC scores in case of MS/MS data, (Tables 3 and 4, row noncross). However, one can conclude that the same factors and factor interactions are significant if comparing PMF and MS/MS data.

### Dissimilarity measures with small variance and high PAUC scores

**Figure 3:**
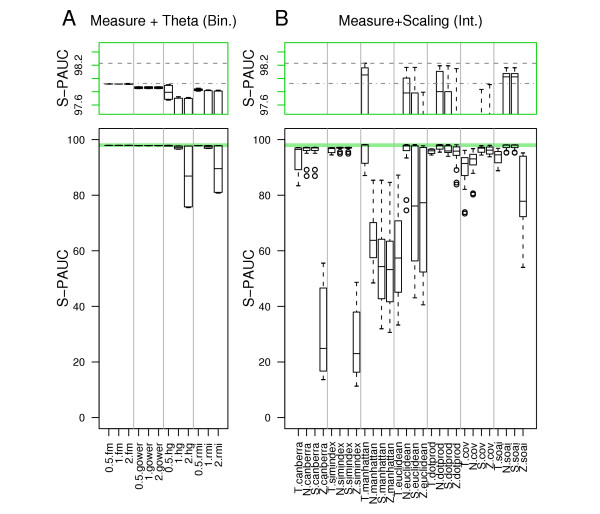
**A**: Boxplot of the sensitivity-PAUC (sensitivity given a FP-rate ∈ [0, 0.1]) itemised according the factors *dissimilarity measure *and *θ *(weighting of non-matching peaks) for the binary measure based peak-list comparisons. **B**: Boxplot of the factors *scale *(cf. Methods – Scaling) and *measure *of the sensitivity-PAUC (sensitivity given a FP-rate ∈ [0, 0.1]) for intensity measure based peak-list comparisons. The **top **panels show a clip (ZOOM) of the bottom boxplot, indicated by the green horizontal line. X-axis labels: fm – Fowlkes-Mallows statistics, gower – Gower coefficients, hg – Huberts Γ, rmi – relative mutual information, canberra – Canberra distance, simindex – similarity index, manhattan – Manhattan distance, euclidean – Euclidean distance, dotprod – dot-product measure, cov – covariance, soai – sum of agreeing intensities. Scaling: T – total ion count, N – vector length, S – root mean square, R – ranks.

The Figure 3 shows the boxplot of the sensitivity PAUCs measure (PMF data) itemised according to the factors explaining the largest variance. These included measure and weight of non matching peaks *θ *(binary measures, Figure 3A) and measure and scaling (intensity based measures, Figure 3B).

**Figure 4:**
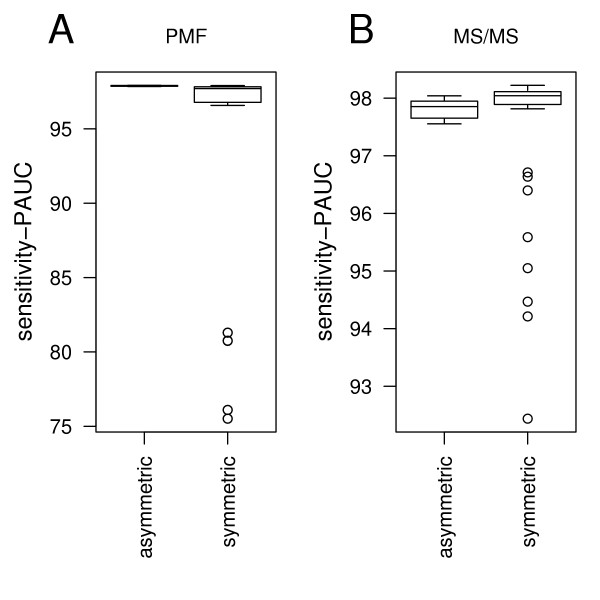
Boxplot **A**: Comparison of the sensitivity-PAUCs (computed for FP-rate ∈ [0, 0.1]) computed for the assymetric binary measures with sensitivity-PAUCs of the symmetric binary measures, in case of the PMF dataset. Boxplot **B**: Comparison of the sensitivity-PAUC (computed for FP-rate ∈ [0, 0.1]) computed for the assymetric binary measures with the sensitivity-PAUCs of the symmetric binary measures, in case of the MS/MS dataset.

In case of binary measures (Figure 3A) the largest PAUC scores were measured for the Fowlkes-Mallows statistics (Figure 3, left panel) followed by the Gower coefficient. Other factors had a negligible impact on the measures as the small height of the boxes indicates. In Figure 4A (PMF data) and 4B (MS/MS data) we compared the scores obtained by the asymmetric binary measures (Fowlkes Mallows statistics and Gower coefficient) with those acquired by the symmetric binary measures (Huberts Gamma and Relative Mutual information). The figure revealed that for MS/MS data, the symmetric measures performed better (right panel). The conclusion, which can be drawn from this observation is that for MS/MS data a lack of peaks at given masses was more significant than for PMF data. This is in agreement with the higher peak density of MS/MS peak-lists.

The boxplot (Figure 3B) demonstrates that the PAUC scores computed using the Manhattan and Euclidean distances, exhibited higher overall variance, than the dot product measure and the sum of agreeing intensities. These measures (Manhattan and Euclidean distances) were influenced by the weighting of non-matching peaks *θ *and peak-list pair length *N *(cf. Methods, Equation 3). These two factors did not influence the outcome of the dot-product measure, which measures only the similarity of matching peaks. However, for a fixed combination of measure and scaling, the Manhattan distance with the total ion count (*l*_1_-norm) scaling and the Euclidean distance with the vector norm (*l*_2_-norm) scaling presented an eminently small variance of the PAUC measure. This reduction of the variance occurred because the factor 'peak-list length' did not influence the outcome of the comparison. Notably good choices of intensity scaling in the case of the sum of agreeing intensities (soai) and the dot product (dotprod) measure were either the vector norm or the root mean square scaling. Remarkably, the widely used dot product measure did not achieved the highest PAUC score (top panel Figure 3B).

The analysis presented here reproduced published results, demonstrating that the relative distances (cf. Methods 12) performed worse than the dot product measure [33]. Furthermore, it identified other measures that performed equally or better than the dot product measure.

### Intensity transformation and ANOVA

**Figure 5:**
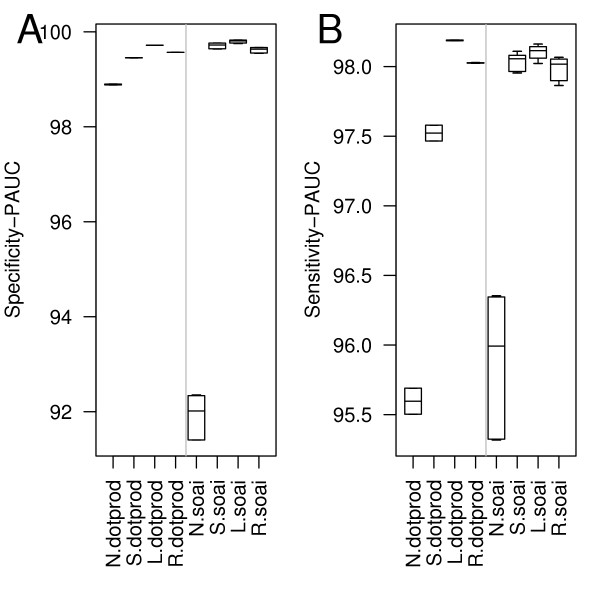
**A**: Boxplot of the specificity-PAUC (specificity given a TP-rate ∈ [0.9, 1]) for the dot-product measure (dotprod) and sum of agreeing intensities (soai). **B **Boxplot of the sensitivity-PAUC (sensitivity given a FP-rate ∈ [0, 0.1]). N – raw intensities, S – square root transformed intensities, L – log transformed intensities, R – intensity ranks.

As the %MSQ scores of the ANOVA analysis reveal, the intensity transformation has a smaller impact on the peak-list comparison process if set in relation with the distance measure and scaling. However, the proper choice of the intensity transformation can increase the PAUC scores. The Boxplot (Figure 5) of the sensitivity-PAUC (left) and specificity-PAUC (right) score, computed using the dot-product and the sum of agreeing intensities measures (both computed on vector length scaled data), shows how the intensity transformation influenced the classification. As predicted by the analysis of variance stabilisation (cf. Appendix – Peak intensity transformation), the log transformation of intensities performed best for both measures.

**Figure 6:**
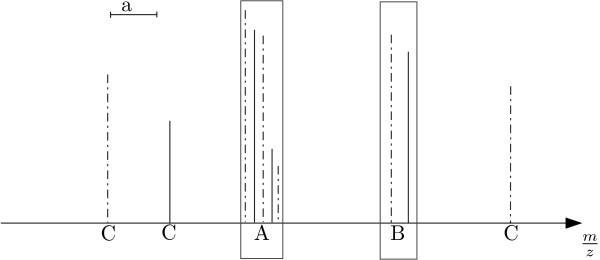
Stick spectrum of two peak-lists X (black lines) and Y (black dot dashed lines). Upper left corner – accuracy of the mass measurement *a*. *A *– ambiguous match of five peaks. *B *– unambiguous match of two peaks. *C *– peaks not matching.

Interestingly, in the case of MS/MS data we did not observe any differences in the PAUC score due to intensity transformation (not shown). This was due to the fact that we were able only, using a dataset where spectra IDs where assigned by database searches, to determine whether a pairwise comparison performed different or equal to the database search algorithms. It means, that the MS/MS database searches did not perform better than the pairwise peak-list comparison computed with the worst intensity transformation.

### The peak-list length

We examined two ways of defining the length of the matched peak-lists, first by setting *N *= 0 in Equation 3 (cf. Methods), and second to a user defined value *N *(*N *= 250 in the case of PMF data and *N *= 400 in case of MS/MS data). The missing peak-pairs were augmented by peaks of zero intensity.

The symmetric binary measures Huberts Γ and relative mutual information significantly interacted with peak-list length (*length *× *measure*) as well as with the weight of non-matching peaks (*θ *× *length*) (see high %MSQ values in Table 3, bottom panel). These interactions were not observed for the asymmetric binary measures, what caused the third order interaction *measure *× *θ *× *length *(Table 3, bottom panel: Final model). In case of PMF and MS/MS data, the best combination of factors for both symmetric binary measures was visible when *N *= 250 or *N *= 400 respectively, and *θ *= 0.5.

In case of the intensity based dissimilarity measures Manhattan and Euclidean distance, a strong interaction between the factor 'peak-list length' and 'measure' (Table 4, bottom panel: Final model row *length *× *measure*) was observed, except for a case when the *L*_1_*metric *(Manhattan distance) was combined with the total ion count (*l *= 1-norm) scaling and the *L*_2 _- *metric *(Euclidean distance) with the *l*_2_-norm (vector length scaling). All the other intensity based measures were practically not influenced by the choice of peak-list length *N *(see Figure 3).

### Differences between binary and intensity based dissimilarities

The dash-dotted line in Figure 3 indicates the maximal sensitivity-PAUC determined for the binary based peak-list comparison, while the dashed line shows the maximal sensitivity-PAUC computed for the intensity based peak-list comparison. If high sensitivities at a high specificity (sensitivity-PAUC) were required, the intensity based peak-list comparison performed better than the binary based peak-list comparison. This is because it is very unlikely that samples from different sources would generate spectra where not only the peak masses, but also the peak intensities were similar. However, if high specificity at high sensitivities was required (PMF data only, not shown), the order reversed and binary measures performed better than intensity based measures. Using binary coding makes it unlikely that peak-lists with matching peaks will generate a large distance because of erroneous peak intensity measurement.

### Weighting of mass measurement accuracy, computing the non-crossing matching and weighting of non-matching peaks

The variance, which is explained by the factor 'non-crossing matching' (cf. Methods – Finding the matching peaks) and 'weight of matching peaks' (cf. Methods – Weighting the missing mass measurement accuracy) was practically zero in case of the PMF data. In case of the MS/MS data, the variance explained by the factor 'weight of matching peaks' and 'non-crossing matching' was small, but not zero.

Using the measures, which take into account the non-matching peaks (e.g. Euclidean distance, Huberts Γ), weighting of mass accuracy may decrease the PAUC obtained by a peak-list comparison. This is because weighting of mass accuracy decreases the weight of matching peak-pairs, but does not affect the non-matching peaks. For example, in the case of the Euclidean distance, which is given by w(a−b)2=(wa2−w2ab+wb2)
 MathType@MTEF@5@5@+=feaafiart1ev1aaatCvAUfKttLearuWrP9MDH5MBPbIqV92AaeXatLxBI9gBaebbnrfifHhDYfgasaacH8akY=wiFfYdH8Gipec8Eeeu0xXdbba9frFj0=OqFfea0dXdd9vqai=hGuQ8kuc9pgc9s8qqaq=dirpe0xb9q8qiLsFr0=vr0=vr0dc8meaabaqaciGacaGaaeqabaqabeGadaaakeaadaGcaaqaaiabdEha3jabcIcaOiabdggaHjabgkHiTiabdkgaIjabcMcaPmaaCaaaleqabaGaeGOmaidaaaqabaGccqGH9aqpdaGcaaqaaiabcIcaOiabdEha3jabdggaHnaaCaaaleqabaGaeGOmaidaaOGaeyOeI0Iaem4DaCNaeGOmaiJaemyyaeMaemOyaiMaey4kaSIaem4DaCNaemOyai2aaWbaaSqabeaacqaIYaGmaaGccqGGPaqkaSqabaaaaa@4610@ weighting of match accuracy may decrease the weight of the term *ab *as well as *b*^2 ^and *a*^2^. For non-matching peaks the term *ab *equals zero, however, the terms *a*^2 ^and *b*^2 ^have a full weight. However, matching peaks have higher discriminating power than non-matching peaks. Thus, exclusively decreasing the contribution of matching peaks decreases the discriminating power of a pairwise peak-list comparison. In order to compensate for the effect of mass measurement accuracy weighting we have introduced the weighting of non-matching peaks by parameter *θ*.

We recommend the usage of both procedures if applying the pairwise peak-list comparison on MS/MS data. Non-crossing matching corrects for errors of the peak extraction procedure. Alternatively, instead of computing the non-crossing matching, the binning of the mass range as described, for example, by Tabb et al. [22] can be used. This however, is limited to data with small mass resolution and a mass range. To decrease the influence of random matches on the dissimilarity, which more frequently occurs in MS/MS peak-lists, the weighting of mass measurement accuracy can be utilised.

## Conclusion

Analysis of variance, based on the factorial structure presented in Table 2 and the PAUC as dependent variable, was used to determine the sensitivities of the factors of pairwise peak-list comparison. To test whether these results apply to different types of mass spectrometric data we used both PMF and MS/MS datasets. The amount of variance explained by the factors was similar for both datasets, which provides evidence that the obtained results might be of general interest.

Two factors, namely measure and intensity scaling and their interactions had the highest impact on the intensity based pairwise peak-list comparison. The combination of the Euclidean distance with vector norm scaling, the Manhattan distance with total ion count scaling and the sum of agreeing intensities with vector length scaling were the best performing measures. A high performing measure with small variance due to the choice of scaling methods was the dot product measure. A further factor, which can be used to increase the classification performance of the peak-list comparison is the intensity transformation with the log function as a best choice. In case of the MS/MS data we recommend to apply the weighting of mass measurement accuracy and combine it with a decrease of the weight of non-matching peaks (*θ *= 0.5), as well as to implement the computation of non-crossing matching.

The most important factors for the comparison of the peak-lists using binary measures are the measure, weight of non-matching peaks (*θ*) and peak-list length *N*. Symmetric measures with large peak-list length *N *and a small weight of non-matching peaks (*θ *= 0.5) performed best for MS/MS data, while asymmetric measures were the most useful during a comparison of PMF data.

A further possible direction to enhance measures of pairwise peak-list dissimilarity would be to combine them with methods that model peak-list properties *i.e. *peptide fragmentation patterns [17].

The pairwise peak-list comparison is a computer model of cluster affiliation, which for a given input of two peak-lists and various control variables such as weight of non-matching peaks generates a single output variable further used to classify the peak-list pair. To conduct the analysis presented here a total number of ≈ 4,000,000,000 pairwise peak-lists comparisons was performed (cf. Methods Computation). In order to reduce a number of required computations and explore a wider range of factors and levels, it might be beneficial to apply different methods to design and analyse computer experiments [47].

The recommended pairwise peak-list comparison approaches can be used as predictive functions of *within *and *between *cluster associations of mass spectrometric peak-list pairs. However, the best value of the discriminatory variable still needs to be determined. This can be achieved, for example, by the use of ROC curves combined with cross validation analysis, but will require a dataset where the identities of the peak-lists are known a-priori.

## Methods

### Datasets and pre-processing

#### PMF-data

The PMF data employed in this study (4532 PMF MS spectra) were derived from three proteome studies. One set contains 1193 PMF MS spectra from bacterial (*Rhodopirellula baltica*) samples (unpublished data). These samples were measured on a Bruker Reflex III reflectron MALDI-TOF MS (Bruker Daltonics, Bremen, Germany). Another set, which contains 1539 PMF spectra from mouse (*Mus musclus*) brain tissue samples (unpublished data) was measured on a Bruker Ultraflex reflectron MALDI-TOF MS (Bruker Daltonics, Bremen, Germany), while the final set, which was measured on an Bruker Autoflex reflectron MALDI-TOF MS (Bruker Daltonics, Bremen, Germany), contains 1800 PMF MS spectra from plant tissue (*Arabidopsis thaliana*) [48,49]. All PMF MS spectra were derived from tryptic protein digests of individually excised protein spots. For this purpose the whole tissue/cell protein extracts of the former mentioned organisms were separated by two-dimensional (2D) gel electrophoresis [48] and visualised with MS compatible Coomassie brilliant blue G250 [49]. The MALDI-TOF MS analysis was performed using delayed ion extraction and employing the MALDI AnchorChipTM targets (Bruker Daltonics, Bremen, Germany). For positively charged ions in the *m/z *range of 700 – 4,500 *m/z *were recorded. Subsequently, the monoisotopic masses of the measured peptides were detected by the SNAP algorithm of the XTOF spectra analysis software (Bruker Daltonics, Bremen, Germany). The sum of the detected monoisotopic masses constitute the raw peak-list (peak-list), which were calibrated to a mass accuracy of 0.05*Da *(or higher) by the in-house developed software mscalib [39,50]. Moreover, the mscalib software was used to filter the peak-lists for irregular peaks that did not follow the general peptide mass rule [12,51]. Additional background peaks (peaks occurring in more than 8% of the spectra [15]) were removed from the peak-lists. The obtained processed peak-lists were then used for the protein database searches with the Mascot search software (Version 1.8.1) [52] employing a mass accuracy of ± 0.1*Da*, setting methionine oxidation as a variable and carbamidomethylation of cysteine residues as fixed modification, and allowing a maximum of 1 missed proteolytic cleavage site. Samples with multiple content/identification were removed from the data. Multiple content of samples was determined by removal of all peaks, matching the highest significant hit in the first search and re-submission of the remaining peaks to a new database search.

#### MS/MS data

To evaluate the distances for the MS/MS data, 70 clusters (spectra assigned to one ID) were randomly chosen (5 replicates obtained) from a large data-set of identified yeast spectra [53]. The protein extraction, sample preparation, measurement and identification were performed as described by Wagner et al. [54]. The analysed MS/MS spectra were recorded on an ESI Ion Trap mass spectrometer (LCQ DECA, Thermo Electron) with the following instrument settings: spray voltage: 1.5 kVolt; data dependent scanning with one full MS spectrum is followed by four independent MS/MS spectra of the four most intensive ions; minimum signal intensity for a peptide to be selected for fragmentation set to 10^6 ^ion counts. These selected and fragmented ions were then excluded from further fragmentation events for 1 minute to prevent repeated MS/MS spectra of identical peptides. The collision energy for the peptide fragmentation was automatically set by the instrument, which was controlled by the Xcalibur software (Versionl.2, Thermo Electron). The post acquisition processing was performed with the Bioworks browser package (Thermo Electron). The resulting peak-lists were automatically stored and assigned to peptide sequences in the yeast protein database [55], by using the Sequest database search algorithm (Version 27) [8,56]. The search parameters employed for the database searches were as follows: a) none or one of the four proteases, as defined by Sickmann et al. [53]; b) mass type: mono isotopic (parent ion and fragment ion) c) amino acid modifications: carbamidomythylated cysteine residues +57*Da *and oxidation on methionine residues +16*Da*, while missed cleavage sites (maximum allowed): 1 missed. We considered spectra identified if they had an *Xcorr *> 2 and an ion coverage of 20%.

### Finding the matching peaks

We considered two peaks *x *and *y *from different peak-lists *X*, *Y *to *match *with an accuracy *a *if |*x *- *y*| <*a *(absolute error) or |x−y|(x+y)/2⋅106<a
 MathType@MTEF@5@5@+=feaafiart1ev1aaatCvAUfKttLearuWrP9MDH5MBPbIqV92AaeXatLxBI9gBaebbnrfifHhDYfgasaacH8akY=wiFfYdH8Gipec8Eeeu0xXdbba9frFj0=OqFfea0dXdd9vqai=hGuQ8kuc9pgc9s8qqaq=dirpe0xb9q8qiLsFr0=vr0=vr0dc8meaabaqaciGacaGaaeqabaqabeGadaaakeaadaWcaaqaaiabcYha8jabdIha4jabgkHiTiabdMha5jabcYha8bqaaiabcIcaOiabdIha4jabgUcaRiabdMha5jabcMcaPiabc+caViabikdaYaaacqGHflY1cqaIXaqmcqaIWaamdaahaaWcbeqaaiabiAda2aaakiabgYda8iabdggaHbaa@42A7@ (relative error in part per million (ppm)). Cases where more than one peak in *Y *match a peak *x *(Figure 6, case A) were resolved by computing a non-crossing matching of the peak-lists. A non-crossing matching a *maximum trace *[57,58] can be computed in time *O*(*n *log *n*), where *n *is the number of peak matches. In our case, we resorted to a simple maximum similarity alignment, which could be banded to improve its *O*(*n*^2^) time complexity. The optimal trace of two sorted mass lists of matching peaks was established by dynamic programming. Let *qual *be a measure of the goodness of the match of two peaks *i.e*.

*qual*_abs _= max{0, *a *- |*x *- *y*|},

where *x *and *y *are masses of peaks from two distinct peak-lists. Then our goal was to maximise the overall quality of all matched peaks. We recorded in the matrix *M*_*i*, *j *_the best possible assignment of the first *i *peaks in the first list and the first *j *peaks in the second list. Hence *M*_*i*,0 _= *M*_*j*,0 _= 0 for all *i*, *j*, and it is easy to see that the following recurrence can be used to derive the overall best assignment:

Mi,j=max⁡{Mi−1,j−1+qualMi−1,jMi,j−1⋅     (1)
 MathType@MTEF@5@5@+=feaafiart1ev1aaatCvAUfKttLearuWrP9MDH5MBPbIqV92AaeXatLxBI9gBaebbnrfifHhDYfgasaacH8akY=wiFfYdH8Gipec8Eeeu0xXdbba9frFj0=OqFfea0dXdd9vqai=hGuQ8kuc9pgc9s8qqaq=dirpe0xb9q8qiLsFr0=vr0=vr0dc8meaabaqaciGacaGaaeqabaqabeGadaaakeaacqWGnbqtdaWgaaWcbaGaemyAaKMaeiilaWIaemOAaOgabeaakiabg2da9iGbc2gaTjabcggaHjabcIha4naaceaaeaqabeaacqWGnbqtdaWgaaWcbaGaemyAaKMaeyOeI0IaeGymaeJaeiilaWIaemOAaOMaeyOeI0IaeGymaedabeaakiabgUcaRiabdghaXjabdwha1jabdggaHjabdYgaSbqaaiabd2eannaaBaaaleaacqWGPbqAcqGHsislcqaIXaqmcqGGSaalcqWGQbGAaeqaaaGcbaGaemyta00aaSbaaSqaaiabdMgaPjabcYcaSiabdQgaQjabgkHiTiabigdaXiabgwSixdqabaaaaOGaay5EaaGaaCzcaiaaxMaadaqadaqaaiabigdaXaGaayjkaiaawMcaaaaa@5AA7@

In this way we could find a non-crossing matching, which minimised the overall errors and unambiguously assigned a peak *x *to a peak *y*.

### Weighting the missing mass measurement accuracy

For computation of the dissimilarities we used the weighting of the mass measurement accuracy [45,46] and the alignment of peak-list by linear regression [12,59]. To model the accuracy of a given match either we weighted the peak intensities in the matching pairs (intensity based measures) or calculated the weight of the match (binary measure) by a triangular function *w*:

wi(xy)={1−|(x−y)|a0 if |(x−y)| <a if |(x−y)| ≥a,     (2)
 MathType@MTEF@5@5@+=feaafiart1ev1aaatCvAUfKttLearuWrP9MDH5MBPbIqV92AaeXatLxBI9gBaebbnrfifHhDYfgasaacH8akY=wiFfYdH8Gipec8Eeeu0xXdbba9frFj0=OqFfea0dXdd9vqai=hGuQ8kuc9pgc9s8qqaq=dirpe0xb9q8qiLsFr0=vr0=vr0dc8meaabaqaciGacaGaaeqabaqabeGadaaakeaacqWG3bWDdaqhaaWcbaGaemyAaKgabaGaeiikaGIaemiEaGNaemyEaKNaeiykaKcaaOGaeyypa0ZaaiqaaqaabeqaaiabigdaXiabgkHiTmaaleaaleaacqGG8baFcqGGOaakcqWG4baEcqGHsislcqWG5bqEcqGGPaqkcqGG8baFaeaacqWGHbqyaaaakeaacqaIWaamaaGaay5EaaqbaeaabiqaaaqaaiabbccaGiadaYyGPbqAcWaGmgOzayMamaiJbccaGiadaYyG8baFcWaGmgikaGIamaiJdIha4jadaYOHsislcWaGmoyEaKNamaiJcMcaPiadaYOG8baFcGaGmIPaVladaYOH8aapcWaGmoyyaegabaGaeeiiaaIamai1bMgaPjadasDGMbGzcWaGuhiiaaIamai1bYha8jadasDGOaakcWaGupiEaGNamai1gkHiTiadas9G5bqEcWaGulykaKIamai1cYha8jacasnMc8Uamai1gwMiZkadas9GHbqycqGGSaalaaGaaCzcaiaaxMaadaqadaqaaiabikdaYaGaayjkaiaawMcaaaaa@89BA@

where *a *is the maximum displacement evaluated and *x *and *y *are peak masses. If the mass difference |*x *- *y*| of two matching peaks increases then the significance of the match is reduced. Before computing the weights, we minimised the overall error of the matching masses by adjusting the two peak-lists using linear regression.

### Non matching peak pairs

Peaks detected in one sample not occurring in the other one (Figure 6, case C), were included in the computation of the dissimilarities. The second peak-list was augmented with a peak of zero intensity, at the mass of the not-matching peak. In addition, the significance of such a peak pair (and peak intensities) could be weighted with the factor *θ*. In this study we examined three values of *θ*, namely 0.5, 1 and 2.

### Binary measures

We have also investigated measures that only use qualitative information in the sense that they evaluate the number of matching and mismatching peaks of both peak-lists. Essentially these measures are numerical functions in the contingency Table 5 derived from both peak-lists. To include the weighting of missing accuracy by the *w *(see Equation 2) and the weighting of non-matching peaks by *θ *we introduced a generalised version of the contingency table. All binary measures introduced below can be computed on the entries of the contingency Table 5.

**Table 5:** Modified contingency table. *M *= max{*N*, *i *+ *θ*·(M01XY
 MathType@MTEF@5@5@+=feaafiart1ev1aaatCvAUfKttLearuWrP9MDH5MBPbIqV92AaeXatLxBI9gBaebbnrfifHhDYfgasaacH8akY=wiFfYdH8Gipec8Eeeu0xXdbba9frFj0=OqFfea0dXdd9vqai=hGuQ8kuc9pgc9s8qqaq=dirpe0xb9q8qiLsFr0=vr0=vr0dc8meaabaqaciGacaGaaeqabaqabeGadaaakeaacqWGnbqtdaqhaaWcbaGaeGimaaJaeGymaedabaGaemiwaGLaemywaKfaaaaa@3250@ + M10XY
 MathType@MTEF@5@5@+=feaafiart1ev1aaatCvAUfKttLearuWrP9MDH5MBPbIqV92AaeXatLxBI9gBaebbnrfifHhDYfgasaacH8akY=wiFfYdH8Gipec8Eeeu0xXdbba9frFj0=OqFfea0dXdd9vqai=hGuQ8kuc9pgc9s8qqaq=dirpe0xb9q8qiLsFr0=vr0=vr0dc8meaabaqaciGacaGaaeqabaqabeGadaaakeaacqWGnbqtdaqhaaWcbaGaeGymaeJaeGimaadabaGaemiwaGLaemywaKfaaaaa@3250@) + M11XY
 MathType@MTEF@5@5@+=feaafiart1ev1aaatCvAUfKttLearuWrP9MDH5MBPbIqV92AaeXatLxBI9gBaebbnrfifHhDYfgasaacH8akY=wiFfYdH8Gipec8Eeeu0xXdbba9frFj0=OqFfea0dXdd9vqai=hGuQ8kuc9pgc9s8qqaq=dirpe0xb9q8qiLsFr0=vr0=vr0dc8meaabaqaciGacaGaaeqabaqabeGadaaakeaacqWGnbqtdaqhaaWcbaGaeGymaeJaeGymaedabaGaemiwaGLaemywaKfaaaaa@3252@} with *N *defined by the user and *c *= 1 in case of Hubert's Gamma or *c *= 0 otherwise.

		object *X*		
		x = 0	x = 1	
object *Y*	y = 0	M00XY MathType@MTEF@5@5@+=feaafiart1ev1aaatCvAUfKttLearuWrP9MDH5MBPbIqV92AaeXatLxBI9gBaebbnrfifHhDYfgasaacH8akY=wiFfYdH8Gipec8Eeeu0xXdbba9frFj0=OqFfea0dXdd9vqai=hGuQ8kuc9pgc9s8qqaq=dirpe0xb9q8qiLsFr0=vr0=vr0dc8meaabaqaciGacaGaaeqabaqabeGadaaakeaacqWGnbqtdaqhaaWcbaGaeGimaaJaeGimaadabaGaemiwaGLaemywaKfaaaaa@324E@	θ⋅M10XY MathType@MTEF@5@5@+=feaafiart1ev1aaatCvAUfKttLearuWrP9MDH5MBPbIqV92AaeXatLxBI9gBaebbnrfifHhDYfgasaacH8akY=wiFfYdH8Gipec8Eeeu0xXdbba9frFj0=OqFfea0dXdd9vqai=hGuQ8kuc9pgc9s8qqaq=dirpe0xb9q8qiLsFr0=vr0=vr0dc8meaabaqaciGacaGaaeqabaqabeGadaaakeaacqaH4oqCcqGHflY1cqWGnbqtdaqhaaWcbaGaeGymaeJaeGimaadabaGaemiwaGLaemywaKfaaaaa@3650@	M0Y MathType@MTEF@5@5@+=feaafiart1ev1aaatCvAUfKttLearuWrP9MDH5MBPbIqV92AaeXatLxBI9gBaebbnrfifHhDYfgasaacH8akY=wiFfYdH8Gipec8Eeeu0xXdbba9frFj0=OqFfea0dXdd9vqai=hGuQ8kuc9pgc9s8qqaq=dirpe0xb9q8qiLsFr0=vr0=vr0dc8meaabaqaciGacaGaaeqabaqabeGadaaakeaacqWGnbqtdaqhaaWcbaGaeGimaadabaGaemywaKfaaaaa@3027@
	y = 1	θ⋅M01XY MathType@MTEF@5@5@+=feaafiart1ev1aaatCvAUfKttLearuWrP9MDH5MBPbIqV92AaeXatLxBI9gBaebbnrfifHhDYfgasaacH8akY=wiFfYdH8Gipec8Eeeu0xXdbba9frFj0=OqFfea0dXdd9vqai=hGuQ8kuc9pgc9s8qqaq=dirpe0xb9q8qiLsFr0=vr0=vr0dc8meaabaqaciGacaGaaeqabaqabeGadaaakeaacqaH4oqCcqGHflY1cqWGnbqtdaqhaaWcbaGaeGimaaJaeGymaedabaGaemiwaGLaemywaKfaaaaa@3650@	M11XY=∑i=0nwixy MathType@MTEF@5@5@+=feaafiart1ev1aaatCvAUfKttLearuWrP9MDH5MBPbIqV92AaeXatLxBI9gBaebbnrfifHhDYfgasaacH8akY=wiFfYdH8Gipec8Eeeu0dXdbba9frFj0=OqFfea0dXdd9vqai=hGuQ8kuc9pgc9s8qqaq=dirpe0xb9q8qiLsFr0=vr0=vr0dc8meaabaqaciGacaGaaeqabaqabeGadaaakeaacqWGnbqtdaqhaaWcbaGaeGymaeJaeGymaedabaGaemiwaGLaemywaKfaaOGaeyypa0ZaaabmaeaacqWG3bWDdaqhaaWcbaGaemyAaKgabaGaemiEaGNaemyEaKhaaaqaaiabdMgaPjabg2da9iabicdaWaqaaiabd6gaUbqdcqGHris5aaaa@3FFB@	M1Y MathType@MTEF@5@5@+=feaafiart1ev1aaatCvAUfKttLearuWrP9MDH5MBPbIqV92AaeXatLxBI9gBaebbnrfifHhDYfgasaacH8akY=wiFfYdH8Gipec8Eeeu0xXdbba9frFj0=OqFfea0dXdd9vqai=hGuQ8kuc9pgc9s8qqaq=dirpe0xb9q8qiLsFr0=vr0=vr0dc8meaabaqaciGacaGaaeqabaqabeGadaaakeaacqWGnbqtdaqhaaWcbaGaeGymaedabaGaemywaKfaaaaa@3029@
		M0X MathType@MTEF@5@5@+=feaafiart1ev1aaatCvAUfKttLearuWrP9MDH5MBPbIqV92AaeXatLxBI9gBaebbnrfifHhDYfgasaacH8akY=wiFfYdH8Gipec8Eeeu0xXdbba9frFj0=OqFfea0dXdd9vqai=hGuQ8kuc9pgc9s8qqaq=dirpe0xb9q8qiLsFr0=vr0=vr0dc8meaabaqaciGacaGaaeqabaqabeGadaaakeaacqWGnbqtdaqhaaWcbaGaeGimaadabaGaemiwaGfaaaaa@3025@	M1X MathType@MTEF@5@5@+=feaafiart1ev1aaatCvAUfKttLearuWrP9MDH5MBPbIqV92AaeXatLxBI9gBaebbnrfifHhDYfgasaacH8akY=wiFfYdH8Gipec8Eeeu0xXdbba9frFj0=OqFfea0dXdd9vqai=hGuQ8kuc9pgc9s8qqaq=dirpe0xb9q8qiLsFr0=vr0=vr0dc8meaabaqaciGacaGaaeqabaqabeGadaaakeaacqWGnbqtdaqhaaWcbaGaeGymaedabaGaemiwaGfaaaaa@3027@	*M*

Peaks present in list *X*, but not in list *Y*, are denoted by M10XY
 MathType@MTEF@5@5@+=feaafiart1ev1aaatCvAUfKttLearuWrP9MDH5MBPbIqV92AaeXatLxBI9gBaebbnrfifHhDYfgasaacH8akY=wiFfYdH8Gipec8Eeeu0xXdbba9frFj0=OqFfea0dXdd9vqai=hGuQ8kuc9pgc9s8qqaq=dirpe0xb9q8qiLsFr0=vr0=vr0dc8meaabaqaciGacaGaaeqabaqabeGadaaakeaacqWGnbqtdaqhaaWcbaGaeGymaeJaeGimaadabaGaemiwaGLaemywaKfaaaaa@3250@, likewise present in *Y*, but not in *X *by M01XY
 MathType@MTEF@5@5@+=feaafiart1ev1aaatCvAUfKttLearuWrP9MDH5MBPbIqV92AaeXatLxBI9gBaebbnrfifHhDYfgasaacH8akY=wiFfYdH8Gipec8Eeeu0xXdbba9frFj0=OqFfea0dXdd9vqai=hGuQ8kuc9pgc9s8qqaq=dirpe0xb9q8qiLsFr0=vr0=vr0dc8meaabaqaciGacaGaaeqabaqabeGadaaakeaacqWGnbqtdaqhaaWcbaGaeGimaaJaeGymaedabaGaemiwaGLaemywaKfaaaaa@3250@. We multiplied the mismatches by *θ *to assign a variable weight. Therefore, M10XY
 MathType@MTEF@5@5@+=feaafiart1ev1aaatCvAUfKttLearuWrP9MDH5MBPbIqV92AaeXatLxBI9gBaebbnrfifHhDYfgasaacH8akY=wiFfYdH8Gipec8Eeeu0xXdbba9frFj0=OqFfea0dXdd9vqai=hGuQ8kuc9pgc9s8qqaq=dirpe0xb9q8qiLsFr0=vr0=vr0dc8meaabaqaciGacaGaaeqabaqabeGadaaakeaacqWGnbqtdaqhaaWcbaGaeGymaeJaeGimaadabaGaemiwaGLaemywaKfaaaaa@3250@, as well as M01XY
 MathType@MTEF@5@5@+=feaafiart1ev1aaatCvAUfKttLearuWrP9MDH5MBPbIqV92AaeXatLxBI9gBaebbnrfifHhDYfgasaacH8akY=wiFfYdH8Gipec8Eeeu0xXdbba9frFj0=OqFfea0dXdd9vqai=hGuQ8kuc9pgc9s8qqaq=dirpe0xb9q8qiLsFr0=vr0=vr0dc8meaabaqaciGacaGaaeqabaqabeGadaaakeaacqWGnbqtdaqhaaWcbaGaeGimaaJaeGymaedabaGaemiwaGLaemywaKfaaaaa@3250@ were replaced by θ⋅M10XY
 MathType@MTEF@5@5@+=feaafiart1ev1aaatCvAUfKttLearuWrP9MDH5MBPbIqV92AaeXatLxBI9gBaebbnrfifHhDYfgasaacH8akY=wiFfYdH8Gipec8Eeeu0xXdbba9frFj0=OqFfea0dXdd9vqai=hGuQ8kuc9pgc9s8qqaq=dirpe0xb9q8qiLsFr0=vr0=vr0dc8meaabaqaciGacaGaaeqabaqabeGadaaakeaacqaH4oqCcqGHflY1cqWGnbqtdaqhaaWcbaGaeGymaeJaeGimaadabaGaemiwaGLaemywaKfaaaaa@3650@ and θ⋅M01XY
 MathType@MTEF@5@5@+=feaafiart1ev1aaatCvAUfKttLearuWrP9MDH5MBPbIqV92AaeXatLxBI9gBaebbnrfifHhDYfgasaacH8akY=wiFfYdH8Gipec8Eeeu0xXdbba9frFj0=OqFfea0dXdd9vqai=hGuQ8kuc9pgc9s8qqaq=dirpe0xb9q8qiLsFr0=vr0=vr0dc8meaabaqaciGacaGaaeqabaqabeGadaaakeaacqaH4oqCcqGHflY1cqWGnbqtdaqhaaWcbaGaeGimaaJaeGymaedabaGaemiwaGLaemywaKfaaaaa@3650@, respectively. To include the weighting of missing mass accuracy in computing the dissimilarities one can set M11XY=∑i=0nwixy
 MathType@MTEF@5@5@+=feaafiart1ev1aaatCvAUfKttLearuWrP9MDH5MBPbIqV92AaeXatLxBI9gBaebbnrfifHhDYfgasaacH8akY=wiFfYdH8Gipec8Eeeu0dXdbba9frFj0=OqFfea0dXdd9vqai=hGuQ8kuc9pgc9s8qqaq=dirpe0xb9q8qiLsFr0=vr0=vr0dc8meaabaqaciGacaGaaeqabaqabeGadaaakeaacqWGnbqtdaqhaaWcbaGaeGymaeJaeGymaedabaGaemiwaGLaemywaKfaaOGaeyypa0ZaaabmaeaacqWG3bWDdaqhaaWcbaGaemyAaKgabaGaemiEaGNaemyEaKhaaaqaaiabdMgaPjabg2da9iabicdaWaqaaiabd6gaUbqdcqGHris5aaaa@3FFB@, with wixy
 MathType@MTEF@5@5@+=feaafiart1ev1aaatCvAUfKttLearuWrP9MDH5MBPbIqV92AaeXatLxBI9gBaebbnrfifHhDYfgasaacH8akY=wiFfYdH8Gipec8Eeeu0xXdbba9frFj0=OqFfea0dXdd9vqai=hGuQ8kuc9pgc9s8qqaq=dirpe0xb9q8qiLsFr0=vr0=vr0dc8meaabaqaciGacaGaaeqabaqabeGadaaakeaacqWG3bWDdaqhaaWcbaGaemyAaKgabaGaemiEaGNaemyEaKhaaaaa@32A1@ defined by the Equation 2. Our data are asymmetric in the sense that we can only evaluate existing peaks and do not count the absence of peaks in both peak-lists at a mass. Measures that utilise only this information are the Gower coefficient and Fowlkes-Mallows statistics. Additionally, we were interested in the performance of measures that take into account the marginal *M *and hence the entry M00XY
 MathType@MTEF@5@5@+=feaafiart1ev1aaatCvAUfKttLearuWrP9MDH5MBPbIqV92AaeXatLxBI9gBaebbnrfifHhDYfgasaacH8akY=wiFfYdH8Gipec8Eeeu0xXdbba9frFj0=OqFfea0dXdd9vqai=hGuQ8kuc9pgc9s8qqaq=dirpe0xb9q8qiLsFr0=vr0=vr0dc8meaabaqaciGacaGaaeqabaqabeGadaaakeaacqWGnbqtdaqhaaWcbaGaeGimaaJaeGimaadabaGaemiwaGLaemywaKfaaaaa@324E@ is required (Hubert's Γ (Appendix Equation 16) or the relative mutual information (Appendix Equation 19)).

Since the peak-lists can have different length and the maximal peak-list length is undefined, we defined the entry *M *length of a matched peak-lists pairs as follows:

M=max⁡{N,c+θ⋅(M01XY+M10XY)+M11XY},     (3)
 MathType@MTEF@5@5@+=feaafiart1ev1aaatCvAUfKttLearuWrP9MDH5MBPbIqV92AaeXatLxBI9gBaebbnrfifHhDYfgasaacH8akY=wiFfYdH8Gipec8Eeeu0xXdbba9frFj0=OqFfea0dXdd9vqai=hGuQ8kuc9pgc9s8qqaq=dirpe0xb9q8qiLsFr0=vr0=vr0dc8meaabaqaciGacaGaaeqabaqabeGadaaakeaacqWGnbqtcqGH9aqpcyGGTbqBcqGGHbqycqGG4baEcqGG7bWEcqWGobGtcqGGSaalcqWGJbWycqGHRaWkcqaH4oqCcqGHflY1cqGGOaakcqWGnbqtdaqhaaWcbaGaeGimaaJaeGymaedabaGaemiwaGLaemywaKfaaOGaey4kaSIaemyta00aa0baaSqaaiabigdaXiabicdaWaqaaiabdIfayjabdMfazbaakiabcMcaPiabgUcaRiabd2eannaaDaaaleaacqaIXaqmcqaIXaqmaeaacqWGybawcqWGzbqwaaGccqGG9bqFcqGGSaalcaWLjaGaaCzcamaabmaabaGaeG4mamdacaGLOaGaayzkaaaaaa@5750@

where *N *is an arbitrary user defined constant and *c *= 1 in case of the Huberts Γ and *c *= 0 otherwise. By this definition, due to the use of the maximum function we avoided the case when M00XY
 MathType@MTEF@5@5@+=feaafiart1ev1aaatCvAUfKttLearuWrP9MDH5MBPbIqV92AaeXatLxBI9gBaebbnrfifHhDYfgasaacH8akY=wiFfYdH8Gipec8Eeeu0xXdbba9frFj0=OqFfea0dXdd9vqai=hGuQ8kuc9pgc9s8qqaq=dirpe0xb9q8qiLsFr0=vr0=vr0dc8meaabaqaciGacaGaaeqabaqabeGadaaakeaacqWGnbqtdaqhaaWcbaGaeGimaaJaeGimaadabaGaemiwaGLaemywaKfaaaaa@324E@ becomes less than zero (see equation 4 for definition of M00XY
 MathType@MTEF@5@5@+=feaafiart1ev1aaatCvAUfKttLearuWrP9MDH5MBPbIqV92AaeXatLxBI9gBaebbnrfifHhDYfgasaacH8akY=wiFfYdH8Gipec8Eeeu0xXdbba9frFj0=OqFfea0dXdd9vqai=hGuQ8kuc9pgc9s8qqaq=dirpe0xb9q8qiLsFr0=vr0=vr0dc8meaabaqaciGacaGaaeqabaqabeGadaaakeaacqWGnbqtdaqhaaWcbaGaeGimaaJaeGimaadabaGaemiwaGLaemywaKfaaaaa@324E@). In this study we used two different values of *N*. We set *N *= 0 and the second value equal to twice the length of the longest peak-list in each dataset.

Given all entries of the modified contingency Table (5), the marginals could be computed by equations (4 – 8):

M00XY=M−(θ⋅M01XY+θ⋅M10XY+∑wixy),     (4)
 MathType@MTEF@5@5@+=feaafiart1ev1aaatCvAUfKttLearuWrP9MDH5MBPbIqV92AaeXatLxBI9gBaebbnrfifHhDYfgasaacH8akY=wiFfYdH8Gipec8Eeeu0xXdbba9frFj0=OqFfea0dXdd9vqai=hGuQ8kuc9pgc9s8qqaq=dirpe0xb9q8qiLsFr0=vr0=vr0dc8meaabaqaciGacaGaaeqabaqabeGadaaakeaacqWGnbqtdaqhaaWcbaGaeGimaaJaeGimaadabaGaemiwaGLaemywaKfaaOGaeyypa0Jaemyta0KaeyOeI0IaeiikaGIaeqiUdeNaeyyXICTaemyta00aa0baaSqaaiabicdaWiabigdaXaqaaiabdIfayjabdMfazbaakiabgUcaRiabeI7aXjabgwSixlabd2eannaaDaaaleaacqaIXaqmcqaIWaamaeaacqWGybawcqWGzbqwaaGccqGHRaWkdaaeabqaaiabdEha3naaDaaaleaacqWGPbqAaeaacqWG4baEcqWG5bqEaaaabeqab0GaeyyeIuoakiabcMcaPiabcYcaSiaaxMaacaWLjaWaaeWaaeaacqaI0aanaiaawIcacaGLPaaaaaa@58E5@

M1X=θ⋅M10XY+∑wixy,     (5)
 MathType@MTEF@5@5@+=feaafiart1ev1aaatCvAUfKttLearuWrP9MDH5MBPbIqV92AaeXatLxBI9gBaebbnrfifHhDYfgasaacH8akY=wiFfYdH8Gipec8Eeeu0xXdbba9frFj0=OqFfea0dXdd9vqai=hGuQ8kuc9pgc9s8qqaq=dirpe0xb9q8qiLsFr0=vr0=vr0dc8meaabaqaciGacaGaaeqabaqabeGadaaakeaacqWGnbqtdaqhaaWcbaGaeGymaedabaGaemiwaGfaaOGaeyypa0JaeqiUdeNaeyyXICTaemyta00aa0baaSqaaiabigdaXiabicdaWaqaaiabdIfayjabdMfazbaakiabgUcaRmaaqaeabaGaem4DaC3aa0baaSqaaiabdMgaPbqaaiabdIha4jabdMha5baaaeqabeqdcqGHris5aOGaeiilaWIaaCzcaiaaxMaadaqadaqaaiabiwda1aGaayjkaiaawMcaaaaa@4870@

M1Y=θ⋅M01XY+∑wixy,     (6)
 MathType@MTEF@5@5@+=feaafiart1ev1aaatCvAUfKttLearuWrP9MDH5MBPbIqV92AaeXatLxBI9gBaebbnrfifHhDYfgasaacH8akY=wiFfYdH8Gipec8Eeeu0xXdbba9frFj0=OqFfea0dXdd9vqai=hGuQ8kuc9pgc9s8qqaq=dirpe0xb9q8qiLsFr0=vr0=vr0dc8meaabaqaciGacaGaaeqabaqabeGadaaakeaacqWGnbqtdaqhaaWcbaGaeGymaedabaGaemywaKfaaOGaeyypa0JaeqiUdeNaeyyXICTaemyta00aa0baaSqaaiabicdaWiabigdaXaqaaiabdIfayjabdMfazbaakiabgUcaRmaaqaeabaGaem4DaC3aa0baaSqaaiabdMgaPbqaaiabdIha4jabdMha5baakiabcYcaSiaaxMaacaWLjaWaaeWaaeaacqaI2aGnaiaawIcacaGLPaaaaSqabeqaniabggHiLdaaaa@487F@

M0X=θ⋅M01XY+M00XY, and     (7)
 MathType@MTEF@5@5@+=feaafiart1ev1aaatCvAUfKttLearuWrP9MDH5MBPbIqV92AaeXatLxBI9gBaebbnrfifHhDYfgasaacH8akY=wiFfYdH8Gipec8Eeeu0xXdbba9frFj0=OqFfea0dXdd9vqai=hGuQ8kuc9pgc9s8qqaq=dirpe0xb9q8qiLsFr0=vr0=vr0dc8meaabaqaciGacaGaaeqabaqabeGadaaakeaacqWGnbqtdaqhaaWcbaGaeGimaadabaGaemiwaGfaaOGaeyypa0JaeqiUdeNaeyyXICTaemyta00aa0baaSqaaiabicdaWiabigdaXaqaaiabdIfayjabdMfazbaakiabgUcaRiabd2eannaaDaaaleaacqaIWaamcqaIWaamaeaacqWGybawcqWGzbqwaaGccqGGSaalcqqGGaaicqqGHbqycqqGUbGBcqqGKbazcaWLjaGaaCzcamaabmaabaGaee4naCdacaGLOaGaayzkaaaaaa@4AD1@

M0Y=θ⋅M10XY+M00XY.     (8)
 MathType@MTEF@5@5@+=feaafiart1ev1aaatCvAUfKttLearuWrP9MDH5MBPbIqV92AaeXatLxBI9gBaebbnrfifHhDYfgasaacH8akY=wiFfYdH8Gipec8Eeeu0xXdbba9frFj0=OqFfea0dXdd9vqai=hGuQ8kuc9pgc9s8qqaq=dirpe0xb9q8qiLsFr0=vr0=vr0dc8meaabaqaciGacaGaaeqabaqabeGadaaakeaacqWGnbqtdaqhaaWcbaGaeGimaadabaGaemywaKfaaOGaeyypa0JaeqiUdeNaeyyXICTaemyta00aa0baaSqaaiabigdaXiabicdaWaqaaiabdIfayjabdMfazbaakiabgUcaRiabd2eannaaDaaaleaacqaIWaamcqaIWaamaeaacqWGybawcqWGzbqwaaGccqGGUaGlcaWLjaGaaCzcamaabmaabaGaeGioaGdacaGLOaGaayzkaaaaaa@461E@

A summary of the measures studied here can be found in the the Appendix section.

### Measures based on peak intensities and intensity ranks

Before computing the measures based on peak intensities, we have applied intensity transformations and scaling procedures. The peak intensities were transformed by taking the square root, as suggested by Tabb et al. [22], and the logarithm. Furthermore, we replaced the intensities by their ranks within the peak-list [27]. The scaling procedures used included total ion current count normalisation [34], vector length normalisation, root mean square normalisation and z-score normalisation (a detailed description can be found in the Appendix section).

In our study, we investigated several pairwise similarity measures to compare two peak-lists. These measures are either measures of similarity (such as covariance) or measures of distance (such as the *l*^*p *^metrics). In order to make both classes of measures comparable we transformed each similarity measure into an appropriate dissimilarity measure. Moreover, we introduced the factor *w*_*i *_to weight missing mass measurement accuracy (cf. Methods – Weighting the missing mass measurement accuracy) and non-matching peaks (cf. Methods – Non matching peak pairs).

**The dot-product of two vectors **is defined



where *I*^*X *^and *I*^*Y *^are the intensity vectors of two matched peak-lists (cf. Methods – Finding the matching peaks) of length *N*, and wixy
 MathType@MTEF@5@5@+=feaafiart1ev1aaatCvAUfKttLearuWrP9MDH5MBPbIqV92AaeXatLxBI9gBaebbnrfifHhDYfgasaacH8akY=wiFfYdH8Gipec8Eeeu0xXdbba9frFj0=OqFfea0dXdd9vqai=hGuQ8kuc9pgc9s8qqaq=dirpe0xb9q8qiLsFr0=vr0=vr0dc8meaabaqaciGacaGaaeqabaqabeGadaaakeaacqWG3bWDdaqhaaWcbaGaemyAaKgabaGaemiEaGNaemyEaKhaaaaa@32A1@ is defined by the Equation 2 for matching peaks and equals *θ *for non-matching peaks. In case of sum-mean-square, total ion count and vector length scaling, the product of non matching peak-pairs is zero and therefore this measure is independent of *θ*. If the intensities of the matched peak-lists are *z*-score scaled the outcome will depend on the value of *θ*. Furthermore, augmenting the peak-lists by zero pairs in order to increase their length will increase *DP *for *z*-score and root-mean-square scaled data. The most prominent representative of this family is the spectral angle (the dot-product of vector length normalised data). It has a geometric interpretation. It is equal to the *cosine *of the angle enclosed by the two vectors.

#### Covariance

The covariance is a measure of dependency between random variables *I*^*x *^and *I*^*y *^[61] and is defined as:

Cov(Ix,Iy)=∑i=1nwixy(Iix−I¯x)(Iiy−I¯y)n−1,     (10)
 MathType@MTEF@5@5@+=feaafiart1ev1aaatCvAUfKttLearuWrP9MDH5MBPbIqV92AaeXatLxBI9gBaebbnrfifHhDYfgasaacH8akY=wiFfYdH8Gipec8Eeeu0dXdbba9frFj0=OqFfea0dXdd9vqai=hGuQ8kuc9pgc9s8qqaq=dirpe0xb9q8qiLsFr0=vr0=vr0dc8meaabaqaciGacaGaaeqabaqabeGadaaakeaatCvAUfeBSjuyZL2yd9gzLbvyNv2CaeHbwvMCKfMBHbaceaGaa83qaiaa=9gacaWF2bGaeiikaGIaemysaK0aaWbaaSqabeaacqWG4baEaaGccqGGSaalcqWGjbqsdaahaaWcbeqaaiabdMha5baakiabcMcaPiabg2da9maalaaabaWaaabmaeaacqWG3bWDdaqhaaWcbaGaemyAaKgabaGaemiEaGNaemyEaKhaaOGaeiikaGIaemysaK0aa0baaSqaaiabdMgaPbqaaiabdIha4baakiabgkHiTiqbdMeajzaaraWaaWbaaSqabeaacqWG4baEaaGccqGGPaqkcqGGOaakcqWGjbqsdaqhaaWcbaGaemyAaKgabaGaemyEaKhaaOGaeyOeI0IafmysaKKbaebadaahaaWcbeqaaiabdMha5baakiabcMcaPaWcbaGaemyAaKMaeyypa0JaeGymaedabaGaemOBa4ganiabggHiLdaakeaacqWGUbGBcqGHsislcqaIXaqmaaGaeiilaWIaaCzcaiaaxMaadaqadaqaaiabigdaXiabicdaWaGaayjkaiaawMcaaaaa@6BFF@

where *I*^*X*^, *I*^*Y*^, *N*, wixy
 MathType@MTEF@5@5@+=feaafiart1ev1aaatCvAUfKttLearuWrP9MDH5MBPbIqV92AaeXatLxBI9gBaebbnrfifHhDYfgasaacH8akY=wiFfYdH8Gipec8Eeeu0xXdbba9frFj0=OqFfea0dXdd9vqai=hGuQ8kuc9pgc9s8qqaq=dirpe0xb9q8qiLsFr0=vr0=vr0dc8meaabaqaciGacaGaaeqabaqabeGadaaakeaacqWG3bWDdaqhaaWcbaGaemyAaKgabaGaemiEaGNaemyEaKhaaaaa@32A1@ are defined as above.

The best known representative of this family of measures is the Pearson correlation, which is obtained if we compute the covariance of *z*-score scaled intensity vectors.

#### Metric-based measures

The Euclidean and Manhattan distances belong to the family of *l*^*p *^metrics and can be expressed using equation:

D(Ix,Iy)=(∑i=1Nwixy|Iix−Iiy|p)1/p.     (11)
 MathType@MTEF@5@5@+=feaafiart1ev1aaatCvAUfKttLearuWrP9MDH5MBPbIqV92AaeXatLxBI9gBaebbnrfifHhDYfgasaacH8akY=wiFfYdH8Gipec8Eeeu0xXdbba9frFj0=OqFfea0dXdd9vqai=hGuQ8kuc9pgc9s8qqaq=dirpe0xb9q8qiLsFr0=vr0=vr0dc8meaabaqaciGacaGaaeqabaqabeGadaaakeaacqWGebarcqGGOaakcqWGjbqsdaahaaWcbeqaaiabdIha4baakiabcYcaSiabdMeajnaaCaaaleqabaGaemyEaKhaaOGaeiykaKIaeyypa0ZaaeWaaeaadaaeWbqaaiabdEha3naaDaaaleaacqWGPbqAaeaacqWG4baEcqWG5bqEaaGcdaabdaqaaiabdMeajnaaDaaaleaacqWGPbqAaeaacqWG4baEaaGccqGHsislcqWGjbqsdaqhaaWcbaGaemyAaKgabaGaemyEaKhaaaGccaGLhWUaayjcSdWaaWbaaSqabeaacqWGWbaCaaaabaGaemyAaKMaeyypa0JaeGymaedabaGaemOta4eaniabggHiLdaakiaawIcacaGLPaaadaahaaWcbeqaaiabigdaXiabc+caViabdchaWbaakiabc6caUiaaxMaacaWLjaWaaeWaaeaacqaIXaqmcqaIXaqmaiaawIcacaGLPaaaaaa@5C26@

In case of the Euclidean distance *p *= 2, and for the Manhattan distance *p *= 1. The Euclidean distance penalises large intensity differences mores than the Manhattan distance. The outcome of this measure will change due to different sample wise scaling of the intensities. In case of the *z*-score scaling the outcome will depend on the user defined peak-list length *N *(Equation 3).

#### Similarity index and Canberra distance

The Similarity index [33] and Canberra Distance [62] measure the relative distance and can be expressed by the equation:

DR(Ix,Iy)=(∑i=1Nwixy|Iix−IiyIix+Iiy|∑i=1Nwixy)1/p.     (12)
 MathType@MTEF@5@5@+=feaafiart1ev1aaatCvAUfKttLearuWrP9MDH5MBPbIqV92AaeXatLxBI9gBaebbnrfifHhDYfgasaacH8akY=wiFfYdH8Gipec8Eeeu0xXdbba9frFj0=OqFfea0dXdd9vqai=hGuQ8kuc9pgc9s8qqaq=dirpe0xb9q8qiLsFr0=vr0=vr0dc8meaabaqaciGacaGaaeqabaqabeGadaaakeaacqWGebarcqWGsbGucqGGOaakcqWGjbqsdaahaaWcbeqaaiabdIha4baakiabcYcaSiabdMeajnaaCaaaleqabaGaemyEaKhaaOGaeiykaKIaeyypa0ZaaeWaaeaadaWcaaqaamaaqadabaGaem4DaC3aa0baaSqaaiabdMgaPbqaaiabdIha4jabdMha5baakmaaemaabaWaaSqaaSqaaiabdMeajnaaDaaameaacqWGPbqAaeaacqWG4baEaaWccqGHsislcqWGjbqsdaqhaaadbaGaemyAaKgabaGaemyEaKhaaaWcbaGaemysaK0aa0baaWqaaiabdMgaPbqaaiabdIha4baaliabgUcaRiabdMeajnaaDaaameaacqWGPbqAaeaacqWG5bqEaaaaaaGccaGLhWUaayjcSdaaleaacqWGPbqAcqGH9aqpcqaIXaqmaeaacqWGobGta0GaeyyeIuoaaOqaamaaqadabaGaem4DaC3aa0baaSqaaiabdMgaPbqaaiabdIha4jabdMha5baaaeaacqWGPbqAcqGH9aqpcqaIXaqmaeaacqWGobGta0GaeyyeIuoaaaaakiaawIcacaGLPaaadaahaaWcbeqaaiabigdaXiabc+caViabdchaWbaakiabc6caUiaaxMaacaWLjaWaaeWaaeaacqaIXaqmcqaIYaGmaiaawIcacaGLPaaaaaa@7157@

Setting *p *= 2 yields the similarity index, while *p *= 1 results in the Canberra distance. Similarly, as in case of the *l*^*p *^metrics, the similarity index with *p *= 2 will be more influenced by large intensity differences than the Canberra distance.

If the term *x *+ *y *in the denominator equals zero due to *x *= -*y*, with *x *≠ 0 ∨ *y *≠ 0, infinity +∞ is returned [60].

#### Sum of agreeing intensities

The sum of agreeing intensities is defined by the equation:

SOAI(Ix,Iy)=1−∑i=1nwixymax⁡{(Iix+Iiy)/2−|Iix−Iiy|,0}∑i=1nwixy(Iix+Iiy)/2.     (13)
 MathType@MTEF@5@5@+=feaafiart1ev1aaatCvAUfKttLearuWrP9MDH5MBPbIqV92AaeXatLxBI9gBaebbnrfifHhDYfgasaacH8akY=wiFfYdH8Gipec8Eeeu0xXdbba9frFj0=OqFfea0dXdd9vqai=hGuQ8kuc9pgc9s8qqaq=dirpe0xb9q8qiLsFr0=vr0=vr0dc8meaabaqaciGacaGaaeqabaqabeGadaaakeaacqWGtbWucqWGpbWtcqWGbbqqcqWGjbqscqGGOaakcqWGjbqsdaahaaWcbeqaaiabdIha4baakiabcYcaSiabdMeajnaaCaaaleqabaGaemyEaKhaaOGaeiykaKIaeyypa0JaeGymaeJaeyOeI0YaaSaaaeaadaaeWaqaaiabdEha3naaDaaaleaacqWGPbqAaeaacqWG4baEcqWG5bqEaaGccyGGTbqBcqGGHbqycqGG4baEdaGadaqaamaabmaabaGaemysaK0aa0baaSqaaiabdMgaPbqaaiabdIha4baakiabgUcaRiabdMeajnaaDaaaleaacqWGPbqAaeaacqWG5bqEaaaakiaawIcacaGLPaaacqGGVaWlcqaIYaGmcqGHsisldaabdaqaaiabdMeajnaaDaaaleaacqWGPbqAaeaacqWG4baEaaGccqGHsislcqWGjbqsdaqhaaWcbaGaemyAaKgabaGaemyEaKhaaaGccaGLhWUaayjcSdGaeiilaWIaeGimaadacaGL7bGaayzFaaaaleaacqWGPbqAcqGH9aqpcqaIXaqmaeaacqWGUbGBa0GaeyyeIuoaaOqaamaaqadabaGaem4DaC3aa0baaSqaaiabdMgaPbqaaiabdIha4jabdMha5baakmaabmaabaGaemysaK0aa0baaSqaaiabdMgaPbqaaiabdIha4baakiabgUcaRiabdMeajnaaDaaaleaacqWGPbqAaeaacqWG5bqEaaaakiaawIcacaGLPaaacqGGVaWlcqaIYaGmaSqaaiabdMgaPjabg2da9iabigdaXaqaaiabd6gaUbqdcqGHris5aaaakiabc6caUiaaxMaacaWLjaWaaeWaaeaacqaIXaqmcqaIZaWmaiaawIcacaGLPaaaaaa@89EB@

It shares with the similarity index and the Canberra distance the property that each pair of matching peaks will contribute to the final score a proportion in the range of [0,1/*n*]. The sum of agreeing intensities however, puts more emphasis on the agreement of peak intensities. Peak pairs whose intensity differences are larger than their average intensity receive a weight of zero.

### Computation

All scores presented in the results section were computed for 75 clusters. The clusters were sampled from the datasets without replacement. For each cluster we randomly chose 2 – 20 (PMF-data) 2 – 7 (MS/MS) samples. This procedure was repeated five times and the average of the scores was computed. The pairwise peak-list comparison approaches were computed with a mass measurement error of 0.7*Da *for the MS/MS data, and of 0.2*Da *for the PMF data. The PAUC areas were computed using in-house developed R functions. Other R packages provide a huge variety of statistical tools for further analysis of the dissimilarities such as clustering algorithms and validation or multidimensional scaling methods [67].

## Appendix

### Binary measures

#### Jaccard/Gower coefficient

The matching peak count is the dot-product of the two peak-lists and counts the number of matching peaks (M11XY
 MathType@MTEF@5@5@+=feaafiart1ev1aaatCvAUfKttLearuWrP9MDH5MBPbIqV92AaeXatLxBI9gBaebbnrfifHhDYfgasaacH8akY=wiFfYdH8Gipec8Eeeu0xXdbba9frFj0=OqFfea0dXdd9vqai=hGuQ8kuc9pgc9s8qqaq=dirpe0xb9q8qiLsFr0=vr0=vr0dc8meaabaqaciGacaGaaeqabaqabeGadaaakeaacqWGnbqtdaqhaaWcbaGaeGymaeJaeGymaedabaGaemiwaGLaemywaKfaaaaa@3252@). Since peak-lists have different numbers of non-zero elements, this dot product must be normalised by the total counts. The Jaccard coefficient is a normalised version of the matching peak count, whose distance version is given by:

G(X,Y)=M01XY+M10XYM01XY+M10XY+M11XY.     (14)
 MathType@MTEF@5@5@+=feaafiart1ev1aaatCvAUfKttLearuWrP9MDH5MBPbIqV92AaeXatLxBI9gBaebbnrfifHhDYfgasaacH8akY=wiFfYdH8Gipec8Eeeu0xXdbba9frFj0=OqFfea0dXdd9vqai=hGuQ8kuc9pgc9s8qqaq=dirpe0xb9q8qiLsFr0=vr0=vr0dc8meaabaqaciGacaGaaeqabaqabeGadaaakeaacqWGhbWrcqGGOaakcqWGybawcqGGSaalcqWGzbqwcqGGPaqkcqGH9aqpdaWcaaqaaiabd2eannaaDaaaleaacqaIWaamcqaIXaqmaeaacqWGybawcqWGzbqwaaGccqGHRaWkcqWGnbqtdaqhaaWcbaGaeGymaeJaeGimaadabaGaemiwaGLaemywaKfaaaGcbaGaemyta00aa0baaSqaaiabicdaWiabigdaXaqaaiabdIfayjabdMfazbaakiabgUcaRiabd2eannaaDaaaleaacqaIXaqmcqaIWaamaeaacqWGybawcqWGzbqwaaGccqGHRaWkcqWGnbqtdaqhaaWcbaGaeGymaeJaeGymaedabaGaemiwaGLaemywaKfaaaaakiabc6caUiaaxMaacaWLjaWaaeWaaeaacqaIXaqmcqaI0aanaiaawIcacaGLPaaaaaa@587C@

A generalised version of the Jaccard coefficient in which M01XY
 MathType@MTEF@5@5@+=feaafiart1ev1aaatCvAUfKttLearuWrP9MDH5MBPbIqV92AaeXatLxBI9gBaebbnrfifHhDYfgasaacH8akY=wiFfYdH8Gipec8Eeeu0xXdbba9frFj0=OqFfea0dXdd9vqai=hGuQ8kuc9pgc9s8qqaq=dirpe0xb9q8qiLsFr0=vr0=vr0dc8meaabaqaciGacaGaaeqabaqabeGadaaakeaacqWGnbqtdaqhaaWcbaGaeGimaaJaeGymaedabaGaemiwaGLaemywaKfaaaaa@3250@ and M10XY
 MathType@MTEF@5@5@+=feaafiart1ev1aaatCvAUfKttLearuWrP9MDH5MBPbIqV92AaeXatLxBI9gBaebbnrfifHhDYfgasaacH8akY=wiFfYdH8Gipec8Eeeu0xXdbba9frFj0=OqFfea0dXdd9vqai=hGuQ8kuc9pgc9s8qqaq=dirpe0xb9q8qiLsFr0=vr0=vr0dc8meaabaqaciGacaGaaeqabaqabeGadaaakeaacqWGnbqtdaqhaaWcbaGaeGymaeJaeGimaadabaGaemiwaGLaemywaKfaaaaa@3250@ is weighted by a constant *θ *was introduced by Gower et al. [63].

#### Fowlkes-Mallows statistics

The Fowlkes-Mallows statistics [64] (introduced in the context of clustering validation by use of contingency tables) are the matching peak counts normalised by the geometric mean of the peak-lists lengths. The equation of the distance-like version is given by:

FM(X,Y)=M11XYM1X⋅M1Y.     (15)
 MathType@MTEF@5@5@+=feaafiart1ev1aaatCvAUfKttLearuWrP9MDH5MBPbIqV92AaeXatLxBI9gBaebbnrfifHhDYfgasaacH8akY=wiFfYdH8Gipec8Eeeu0xXdbba9frFj0=OqFfea0dXdd9vqai=hGuQ8kuc9pgc9s8qqaq=dirpe0xb9q8qiLsFr0=vr0=vr0dc8meaabaqaciGacaGaaeqabaqabeGadaaakeaacqWGgbGrcqWGnbqtcqGGOaakcqWGybawcqGGSaalcqWGzbqwcqGGPaqkcqGH9aqpdaWcaaqaaiabd2eannaaDaaaleaacqaIXaqmcqaIXaqmaeaacqWGybawcqWGzbqwaaaakeaadaGcaaqaaiabd2eannaaDaaaleaacqaIXaqmaeaacqWGybawaaGccqGHflY1cqWGnbqtdaqhaaWcbaGaeGymaedabaGaemywaKfaaaqabaaaaOGaeiOla4IaaCzcaiaaxMaadaqadaqaaiabigdaXiabiwda1aGaayjkaiaawMcaaaaa@49AB@

#### Huberts Γ

Using binary signals, we can transform the formula of the correlation coefficient such that it uses the values of the contingency table to obtain:

HG(X,Y)=M⋅M11XY−M1X⋅M1YM0X⋅M1X⋅M0Y⋅M1Y.     (16)
 MathType@MTEF@5@5@+=feaafiart1ev1aaatCvAUfKttLearuWrP9MDH5MBPbIqV92AaeXatLxBI9gBaebbnrfifHhDYfgasaacH8akY=wiFfYdH8Gipec8Eeeu0xXdbba9frFj0=OqFfea0dXdd9vqai=hGuQ8kuc9pgc9s8qqaq=dirpe0xb9q8qiLsFr0=vr0=vr0dc8meaabaqaciGacaGaaeqabaqabeGadaaakeaacqWGibascqWGhbWrcqGGOaakcqWGybawcqGGSaalcqWGzbqwcqGGPaqkcqGH9aqpdaWcaaqaaiabd2eanjabgwSixlabd2eannaaDaaaleaacqaIXaqmcqaIXaqmaeaacqWGybawcqWGzbqwaaGccqGHsislcqWGnbqtdaqhaaWcbaGaeGymaedabaGaemiwaGfaaOGaeyyXICTaemyta00aa0baaSqaaiabigdaXaqaaiabdMfazbaaaOqaamaakaaabaGaemyta00aa0baaSqaaiabicdaWaqaaiabdIfaybaakiabgwSixlabd2eannaaDaaaleaacqaIXaqmaeaacqWGybawaaGccqGHflY1cqWGnbqtdaqhaaWcbaGaeGimaadabaGaemywaKfaaOGaeyyXICTaemyta00aa0baaSqaaiabigdaXaqaaiabdMfazbaaaeqaaaaakiabc6caUiaaxMaacaWLjaWaaeWaaeaacqaIXaqmcqaI2aGnaiaawIcacaGLPaaaaaa@62E9@

We observed that the numerator was maximised if all signals were expressed equally. To avoid the fact that the denominator becomes zero (which is the case if M0X
 MathType@MTEF@5@5@+=feaafiart1ev1aaatCvAUfKttLearuWrP9MDH5MBPbIqV92AaeXatLxBI9gBaebbnrfifHhDYfgasaacH8akY=wiFfYdH8Gipec8Eeeu0xXdbba9frFj0=OqFfea0dXdd9vqai=hGuQ8kuc9pgc9s8qqaq=dirpe0xb9q8qiLsFr0=vr0=vr0dc8meaabaqaciGacaGaaeqabaqabeGadaaakeaacqWGnbqtdaqhaaWcbaGaeGimaadabaGaemiwaGfaaaaa@3025@ or M0Y
 MathType@MTEF@5@5@+=feaafiart1ev1aaatCvAUfKttLearuWrP9MDH5MBPbIqV92AaeXatLxBI9gBaebbnrfifHhDYfgasaacH8akY=wiFfYdH8Gipec8Eeeu0xXdbba9frFj0=OqFfea0dXdd9vqai=hGuQ8kuc9pgc9s8qqaq=dirpe0xb9q8qiLsFr0=vr0=vr0dc8meaabaqaciGacaGaaeqabaqabeGadaaakeaacqWGnbqtdaqhaaWcbaGaeGimaadabaGaemywaKfaaaaa@3027@ = 0 and occurs if one peak-list is included in the other) we set *c *= 1 in equation (3).

#### Relative mutual information

We were additionally interested in the performance of information theoretic concepts. Given the two peak-lists, *X *and *Y*, the amount of information about peak-list *X *inherent in peak-list *Y *(and vice versa) is given by the *mutual information *(**H**) [65]:

H(X;Y)=∑i=01∑j=01MijXYMlog⁡2(MijXY⋅MMiX⋅MjY).     (17)
 MathType@MTEF@5@5@+=feaafiart1ev1aaatCvAUfKttLearuWrP9MDH5MBPbIqV92AaeXatLxBI9gBaebbnrfifHhDYfgasaacH8akY=wiFfYdH8Gipec8Eeeu0xXdbba9frFj0=OqFfea0dXdd9vqai=hGuQ8kuc9pgc9s8qqaq=dirpe0xb9q8qiLsFr0=vr0=vr0dc8meaabaqaciGacaGaaeqabaqabeGadaaakeaacqWGibascqGGOaakcqWGybawcqGG7aWocqWGzbqwcqGGPaqkcqGH9aqpdaaeWbqaamaaqahabaWaaSaaaeaacqWGnbqtdaqhaaWcbaGaemyAaKMaemOAaOgabaGaemiwaGLaemywaKfaaaGcbaGaemyta0eaaaWcbaGaemOAaOMaeyypa0JaeGimaadabaGaeGymaedaniabggHiLdGccyGGSbaBcqGGVbWBcqGGNbWzdaWgaaWcbaGaeGOmaidabeaaaeaacqWGPbqAcqGH9aqpcqaIWaamaeaacqaIXaqma0GaeyyeIuoakmaabmaabaWaaSaaaeaacqWGnbqtdaqhaaWcbaGaemyAaKMaemOAaOgabaGaemiwaGLaemywaKfaaOGaeyyXICTaemyta0eabaGaemyta00aa0baaSqaaiabdMgaPbqaaiabdIfaybaakiabgwSixlabd2eannaaDaaaleaacqWGQbGAaeaacqWGzbqwaaaaaaGccaGLOaGaayzkaaGaeiOla4IaaCzcaiaaxMaadaqadaqaaiabigdaXiabiEda3aGaayjkaiaawMcaaaaa@6946@

To be able to use the mutual information as a similarity measure, so it could distinguish positive from negative correlation, we introduced the following scaling term [66]:

Δ={−1if M11XY<(M1Y⋅M1X)/M0if M11XY=(M1Y⋅M1X)/M1otherwise.
 MathType@MTEF@5@5@+=feaafiart1ev1aaatCvAUfKttLearuWrP9MDH5MBPbIqV92AaeXatLxBI9gBaebbnrfifHhDYfgasaacH8akY=wiFfYdH8Gipec8Eeeu0xXdbba9frFj0=OqFfea0dXdd9vqai=hGuQ8kuc9pgc9s8qqaq=dirpe0xb9q8qiLsFr0=vr0=vr0dc8meaabaqaciGacaGaaeqabaqabeGadaaakeaacqGHuoarcqGH9aqpdaGabaqaauaabaqadiaaaeaacqGHsislcqaIXaqmaeaacqqGPbqAcqqGMbGzcqqGGaaicqWGnbqtdaqhaaWcbaGaeGymaeJaeGymaedabaGaemiwaGLaemywaKfaaOGaeyipaWJaeiikaGIaemyta00aa0baaSqaaiabigdaXaqaaiabdMfazbaakiabgwSixlabd2eannaaDaaaleaacqaIXaqmaeaacqWGybawaaGccqGGPaqkcqGGVaWlcqWGnbqtaeaacqaIWaamaeaacqqGPbqAcqqGMbGzcqqGGaaicqWGnbqtdaqhaaWcbaGaeGymaeJaeGymaedabaGaemiwaGLaemywaKfaaOGaeyypa0JaeiikaGIaemyta00aa0baaSqaaiabigdaXaqaaiabdMfazbaakiabgwSixlabd2eannaaDaaaleaacqaIXaqmaeaacqWGybawaaGccqGGPaqkcqGGVaWlcqWGnbqtaeaacqaIXaqmaeaacqqGVbWBcqqG0baDcqqGObaAcqqGLbqzcqqGYbGCcqqG3bWDcqqGPbqAcqqGZbWCcqqGLbqzcqqGUaGlaaaacaGL7baaaaa@6FBE@

Furthermore, we adjusted it for the information inherent in the individual peak-lists. The adjustment was done using the entropy of the individual peak-lists, which for a peak-list *X *is given by:

H(X)=−∑i=01MiXMlog⁡2MiXM.     (18)
 MathType@MTEF@5@5@+=feaafiart1ev1aaatCvAUfKttLearuWrP9MDH5MBPbIqV92AaeXatLxBI9gBaebbnrfifHhDYfgasaacH8akY=wiFfYdH8Gipec8Eeeu0xXdbba9frFj0=OqFfea0dXdd9vqai=hGuQ8kuc9pgc9s8qqaq=dirpe0xb9q8qiLsFr0=vr0=vr0dc8meaabaqaciGacaGaaeqabaqabeGadaaakeaacqWGibascqGGOaakcqWGybawcqGGPaqkcqGH9aqpcqGHsisldaaeWbqaamaalaaabaGaemyta00aa0baaSqaaiabdMgaPbqaaiabdIfaybaaaOqaaiabd2eanbaaaSqaaiabdMgaPjabg2da9iabicdaWaqaaiabigdaXaqdcqGHris5aOGagiiBaWMaei4Ba8Maei4zaC2aaSbaaSqaaiabikdaYaqabaGcdaWcaaqaaiabd2eannaaDaaaleaacqWGPbqAaeaacqWGybawaaaakeaacqWGnbqtaaGaeiOla4IaaCzcaiaaxMaadaqadaqaaiabigdaXiabiIda4aGaayjkaiaawMcaaaaa@4E57@

Thus, we defined the relative mutual information:

RH(X,Y)=Δ2H(X;Y)H(X)+H(Y).     (19)
 MathType@MTEF@5@5@+=feaafiart1ev1aaatCvAUfKttLearuWrP9MDH5MBPbIqV92AaeXatLxBI9gBaebbnrfifHhDYfgasaacH8akY=wiFfYdH8Gipec8Eeeu0xXdbba9frFj0=OqFfea0dXdd9vqai=hGuQ8kuc9pgc9s8qqaq=dirpe0xb9q8qiLsFr0=vr0=vr0dc8meaabaqaciGacaGaaeqabaqabeGadaaakeaacqWGsbGucqWGibascqGGOaakcqWGybawcqGGSaalcqWGzbqwcqGGPaqkcqGH9aqpcqGHuoardaWcaaqaaiabikdaYiabdIeaijabcIcaOiabdIfayjabcUda7iabdMfazjabcMcaPaqaaiabdIeaijabcIcaOiabdIfayjabcMcaPiabgUcaRiabdIeaijabcIcaOiabdMfazjabcMcaPaaacqGGUaGlcaWLjaGaaCzcamaabmaabaGaeGymaeJaeGyoaKdacaGLOaGaayzkaaaaaa@4C33@

The relative mutual information is small if both peak-lists are similar and high if they differ. Since the inequalities

*H*(*X*; *Y*) ≥ 0 and *H*(*X*; *Y*) ≤ min{*H*(*X*), *H*(*Y*)}

holds, this measure is bounded to the interval [-1,1]. The relative mutual information has been introduced before [36], in the context of clustering gene expression data.

### Peak intensity scaling

The purpose of scaling is to allow the comparison of peak-lists with different intensity values *i.e. *due to different scale of the detector used or due to different amount of sample. Since intensities in different peak-lists could have different intensity ranges, we used standard scaling procedures to account for this bias.

• Total ion current count normalisation [34,35] is defined as:

Ii'=Ii∑i=1NIi,     (20)
 MathType@MTEF@5@5@+=feaafiart1ev1aaatCvAUfKttLearuWrP9MDH5MBPbIqV92AaeXatLxBI9gBaebbnrfifHhDYfgasaacH8akY=wiFfYdH8Gipec8Eeeu0xXdbba9frFj0=OqFfea0dXdd9vqai=hGuQ8kuc9pgc9s8qqaq=dirpe0xb9q8qiLsFr0=vr0=vr0dc8meaabaqaciGacaGaaeqabaqabeGadaaakeaacqWGjbqsdaqhaaWcbaGaemyAaKgabaGaei4jaCcaaOGaeyypa0ZaaSaaaeaacqWGjbqsdaWgaaWcbaGaemyAaKgabeaaaOqaamaaqadabaGaemysaK0aaSbaaSqaaiabdMgaPbqabaaabaGaemyAaKMaeyypa0JaeGymaedabaGaemOta4eaniabggHiLdaaaOGaeiilaWIaaCzcaiaaxMaadaqadaqaaiabikdaYiabicdaWaGaayjkaiaawMcaaaaa@4299@

where *I*_*i *_is the intensity of the peak *i *in the peak-list of length *N*. Here, the intensities are divided by the sum of all intensities, so that after scaling the sum of the intensities in each peak-list equals one (∑inI'=1
 MathType@MTEF@5@5@+=feaafiart1ev1aaatCvAUfKttLearuWrP9MDH5MBPbIqV92AaeXatLxBI9gBaebbnrfifHhDYfgasaacH8akY=wiFfYdH8Gipec8Eeeu0xXdbba9frFj0=OqFfea0dXdd9vqai=hGuQ8kuc9pgc9s8qqaq=dirpe0xb9q8qiLsFr0=vr0=vr0dc8meaabaqaciGacaGaaeqabaqabeGadaaakeaadaaeWaqaaiabdMeajjabcEcaNiabg2da9iabigdaXaWcbaGaemyAaKgabaGaemOBa4ganiabggHiLdaaaa@3557@). The total ion count is better known as the *l*_1 _– norm since *I*_*i *_> 0 ∀ *i*

• Vector length normalisation is defined as:

Ii'=Ii∑i=1NIi2.     (21)
 MathType@MTEF@5@5@+=feaafiart1ev1aaatCvAUfKttLearuWrP9MDH5MBPbIqV92AaeXatLxBI9gBaebbnrfifHhDYfgasaacH8akY=wiFfYdH8Gipec8Eeeu0xXdbba9frFj0=OqFfea0dXdd9vqai=hGuQ8kuc9pgc9s8qqaq=dirpe0xb9q8qiLsFr0=vr0=vr0dc8meaabaqaciGacaGaaeqabaqabeGadaaakeaacqWGjbqsdaqhaaWcbaGaemyAaKgabaGaei4jaCcaaOGaeyypa0ZaaSaaaeaacqWGjbqsdaWgaaWcbaGaemyAaKgabeaaaOqaamaakaaabaWaaabmaeaacqWGjbqsdaqhaaWcbaGaemyAaKgabaGaeGOmaidaaaqaaiabdMgaPjabg2da9iabigdaXaqaaiabd6eaobqdcqGHris5aaWcbeaaaaGccqGGUaGlcaWLjaGaaCzcamaabmaabaGaeGOmaiJaeGymaedacaGLOaGaayzkaaaaaa@43AD@

Here, the peak intensities are divided by the *l *= 2-norm of the intensity vector, which causes that the Euclidean length of the vector equals one (∑inI2=1
 MathType@MTEF@5@5@+=feaafiart1ev1aaatCvAUfKttLearuWrP9MDH5MBPbIqV92AaeXatLxBI9gBaebbnrfifHhDYfgasaacH8akY=wiFfYdH8Gipec8Eeeu0xXdbba9frFj0=OqFfea0dXdd9vqai=hGuQ8kuc9pgc9s8qqaq=dirpe0xb9q8qiLsFr0=vr0=vr0dc8meaabaqaciGacaGaaeqabaqabeGadaaakeaadaaeWaqaaiabdMeajnaaCaaaleqabaGaeGOmaidaaOGaeyypa0JaeGymaedaleaacqWGPbqAaeaacqWGUbGBa0GaeyyeIuoaaaa@35AA@).

• Root mean square normalisation is defined as:

Ii'=Ii1N−1∑i=1NIi2.     (22)
 MathType@MTEF@5@5@+=feaafiart1ev1aaatCvAUfKttLearuWrP9MDH5MBPbIqV92AaeXatLxBI9gBaebbnrfifHhDYfgasaacH8akY=wiFfYdH8Gipec8Eeeu0xXdbba9frFj0=OqFfea0dXdd9vqai=hGuQ8kuc9pgc9s8qqaq=dirpe0xb9q8qiLsFr0=vr0=vr0dc8meaabaqaciGacaGaaeqabaqabeGadaaakeaacqWGjbqsdaqhaaWcbaGaemyAaKgabaGaei4jaCcaaOGaeyypa0ZaaSaaaeaacqWGjbqsdaWgaaWcbaGaemyAaKgabeaaaOqaamaakaaabaWaaSqaaSqaaiabigdaXaqaaiabd6eaojabgkHiTiabigdaXaaakmaaqadabaGaemysaK0aa0baaSqaaiabdMgaPbqaaiabikdaYaaaaeaacqWGPbqAcqGH9aqpcqaIXaqmaeaacqWGobGta0GaeyyeIuoaaSqabaaaaOGaeiOla4IaaCzcaiaaxMaadaqadaqaaiabikdaYiabikdaYaGaayjkaiaawMcaaaaa@47C7@

Here, the intensities are divided by their root-mean-square [60].

• *z*-score normalisation is defined as:

Ii'=Ii−I¯SN(I),     (23)
 MathType@MTEF@5@5@+=feaafiart1ev1aaatCvAUfKttLearuWrP9MDH5MBPbIqV92AaeXatLxBI9gBaebbnrfifHhDYfgasaacH8akY=wiFfYdH8Gipec8Eeeu0xXdbba9frFj0=OqFfea0dXdd9vqai=hGuQ8kuc9pgc9s8qqaq=dirpe0xb9q8qiLsFr0=vr0=vr0dc8meaabaqaciGacaGaaeqabaqabeGadaaakeaacqWGjbqsdaqhaaWcbaGaemyAaKgabaGaei4jaCcaaOGaeyypa0ZaaSaaaeaacqWGjbqsdaWgaaWcbaGaemyAaKgabeaakiabgkHiTiqbdMeajzaaraaabaGaem4uam1aaSbaaSqaaiabd6eaobqabaGccqGGOaakcqWGjbqscqGGPaqkaaGaeiilaWIaaCzcaiaaxMaadaqadaqaaiabikdaYiabiodaZaGaayjkaiaawMcaaaaa@40FD@

where *I*_*i*_, *i*, *N *defined as above, *Ī *denotes the average intensity of a peak-list and SN=1N−1∑i=1N(Ii−I¯)2
 MathType@MTEF@5@5@+=feaafiart1ev1aaatCvAUfKttLearuWrP9MDH5MBPbIqV92AaeXatLxBI9gBaebbnrfifHhDYfgasaacH8akY=wiFfYdH8Gipec8Eeeu0xXdbba9frFj0=OqFfea0dXdd9vqai=hGuQ8kuc9pgc9s8qqaq=dirpe0xb9q8qiLsFr0=vr0=vr0dc8meaabaqaciGacaGaaeqabaqabeGadaaakeaacqWGtbWudaWgaaWcbaGaemOta4eabeaakiabg2da9maakaaabaWaaSqaaSqaaiabigdaXaqaaiabd6eaojabgkHiTiabigdaXaaakmaaqadabaGaeiikaGIaemysaK0aaSbaaSqaaiabdMgaPbqabaGccqGHsislcuWGjbqsgaqeaiabcMcaPaWcbaGaemyAaKMaeyypa0JaeGymaedabaGaemOta4eaniabggHiLdGcdaahaaWcbeqaaiabikdaYaaaaeqaaaaa@4285@. Here, two scaling steps are performed, centring and subsequent division by the standard deviation. This causes each scaled peak-list to have an average intensity of zero and a standard deviation of one.

The scaling is preferred if intensities and variance in an arbitrary sample are much higher than in the other samples, which will determine the outcome of the peak-list comparisons. Data transformation was applied before the peak-list matching, whereas data scaling was performed for already matched peak-lists.

### Features of the pairwise peak-list comparison and their properties

#### The number of matches

While the intensities of individual peaks may considerably vary between the spectra, the *m/z *values of fragment ions can be measured with at least the accuracy of a single *m/z *in the majority of mass spectrometers. If the primary fragment ions/peptides in a pair of spectra have the same *m/z *locations, the spectra are judged to result from the same peptide/protein, regardless of their peak intensities. Table 6 (rows one and four) summarises the properties of both: mass measurement error (MME) and the mass measurement range of the peak-lists. It also provides the *five-number summary *and the *mean *of the peak-lists lengths. The observed number of matches for *within *and *between *cluster peak-list pairs is shown in Table 6 (rows two, three, five and six). The theoretical probability of *i *matches if two independent peak-lists of known length, mass measurement range and resolution are compared, can be modelled using the hyper-geometric distribution [40]. In case of PMF data, for peak-lists drawn from *between *clusters, a higher number of matches than expected for independent peak-lists was observed. This difference might be due to incomplete separation of proteins obtained after two-dimensional (2D) gel electrophoresis [41] and because the sequence database entries have different database IDs, even if the protein sequences exhibit a high fraction of sequence identity (*i.e. *protein families).

**Table 6:** Peptide (PMF) peak-list and peptide fragment ions (MS/MS) peak-list properties. MME – mass measurement error. The rows 1 and 4 provide &*five-number summary *and the *mean *of the peak-lists lengths (number of peaks in peak-list) in the dataset. Rows 2,3 (PMF) and 5,6 (MS/MS) provide the *five-number summary *and the *mean *of the number of matches observed if comparing *within *and *between *cluster peak-lists pairs. Min. – minimum, 1st Qu. – first quartile, 3rd Qu. – third quartile, Max. – maximum

	Data	MME [Da]	Mass range [Da]							
			Min.	Max.	Min.	1st Qu.	Median	Mean	3rd Qu.	Max.

1	PMF	±0.1	713	4050	3	17	30	36	50	124
2	matching peaks *between *clusters peak-lists				0	0	0	0.62	1	32
3	matching peaks *within *clusters peak-lists				0	7	12	15.4	21	68
4	MS/MS	±0.5	129	2000	35	97	134	136	170	354
5	matching peaks *between *clusters peak-lists				0	9	15	16	22	94
6	matching peaks *within *clusters peak-lists				8	44	56	57	69	133

**Figure 7:**
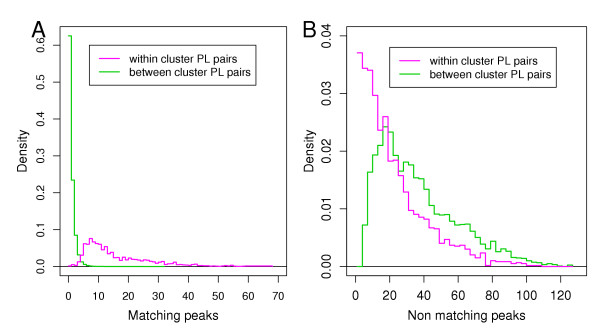
**A **– Histogram of the number (bandwith = 1) of matching peaks for peak-lists chosen from the same cluster (magenta) and from different clusters (green). **B **– Histogram of the number (bandwith = 3) of non-matching peaks, if peak-lists were chosen from the same (magenta) or from different clusters (green).

For 75 clusters of various size (2 – 20 samples/cluster) sampled five times from the PMF dataset, we have computed the number of matching peaks for all peak-list pairs. The number of matching peaks was in almost all cases higher, if the peak-lists compared laid *within *one cluster (magenta histogram, Figure 7A), than if they occurred *between *different clusters (green histogram, Figure 7A). For example, 95% of *within *cluster peak-list pairs had more than 4 matches, but only 1% of *between *cluster peak-list pairs had more than 4 matches. The cases where the number of matches between peak-list from *within *one cluster equalled zero, can be explained by the fact that the spectra were measured on non-overlapping fragments of the same protein.

The masses of randomly matching peaks differ, on average, more than the masses of non-random matching peaks. Therefore, weighting of mass measurement accuracy using a triangular function (see Equation 2) was implemented. This function reduced the weight of peaks with a small overlap.

Furthermore, in case of the MS/MS peak-lists, clusters of peaks separated by a mass smaller than the mass measurement accuracy (which is used for searches of matching peaks) were observed. Therefore, during matching the two peak-lists, some ambiguous matches (that is a peak is assigned to more than one peak in the second peak-list) occurred (cf. Methods – Figure 6, case A). In order to generate an unambiguous pairwise assignment of peaks we computed the non-crossing matching using standard dynamic programming techniques (cf. Methods – Finding the matching peaks).

We concluded that the probability of matches between independent peak-lists is higher in case of MS/MS than PMF data because of its lower mass measurement accuracy, smaller mass range and larger number of peaks. Hence, the number of matches has a lower discriminating power in case of MS/MS than of PMF data.

#### The number of non-matching peaks

To discriminate peak-list pairs as being *within *or *between *clusters the number of non-matching peaks can be used. Figure 7B presents histograms of the number of *non-*matching peaks between peak-list pairs (in magenta – the number of peaks that did not match if we compared two peak-lists *within *a cluster; in green – the number of peaks that did not match if we compare peak-lists pairs *between *two clusters). We observed that the probability of encountering a *within *peak-list pair increased if the number of non-matching peaks was small.

We have evaluated the performance of the following asymmetric binary measures: Gower coefficient [63] (cf. Appendix Equation 14) and Fowlkes-Mallows statistics (cf. Appendix Equation 15). These measures incorporate the number of matches and mismatches. If the length of the aligned peak-lists is defined (see Equation 3), symmetric binary measures *e.g. *Huberts Γ (Appendix Equation 16) and relative mutual information (Appendix Equation 19) can also be used. Furthermore, we examined whether increasing or decreasing the weight of non-matching peaks by a factor of two can increase the performance of the pairwise peak-list comparison (cf. Methods – Non matching peak pairs).

#### Peak intensities

**Figure 8:**
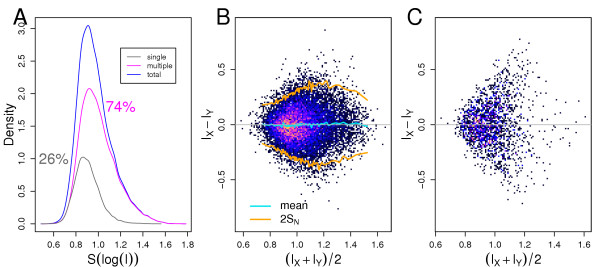
Peak Intensities. **A**) Histogram of intensities: X-axes – Intensity of log transformed root-means-square scaled peak intensities. Y-axis – Frequency. In grey: Histogram of the peak intensities that do **not ***match *a peak in any other peak-lists (peak-lists) *within *the same cluster (this mass is observed only once in the cluster). In magenta: Histogram of intensities of peaks that do *match *a peak within any peak-list *within *cluster (this mass is observed at least twice in the cluster). **B**) Altman Bland plot of intensities of the matching peaks for peak-lists pairs from *within *a cluster. **C**) Altman Bland plot of intensities of matching peaks for peak-lists pairs of *between *clusters.

Intensities associated with the masses observed at least twice *within *a cluster (magenta density, Figure 8A) tend to have higher peak intensities, compared to intensities of peaks whose masses are observed only once *within *a cluster (grey density, Figure 8A). Furthermore, intensities *I*_*X *_and *I*_*Y*_, of matching peaks in peak-lists from *within *a cluster, were more strongly correlated (corPMF(within)(IX,IY)=0.57
 MathType@MTEF@5@5@+=feaafiart1ev1aaatCvAUfKttLearuWrP9MDH5MBPbIqV92AaeXatLxBI9gBaebbnrfifHhDYfgasaacH8akY=wiFfYdH8Gipec8Eeeu0xXdbba9frFj0=OqFfea0dXdd9vqai=hGuQ8kuc9pgc9s8qqaq=dirpe0xb9q8qiLsFr0=vr0=vr0dc8meaabaqaciGacaGaaeqabaqabeGadaaakeaaieaacqWFJbWycqWFVbWBcqWFYbGCdaqhaaWcbaGaemiuaaLaemyta0KaemOrayeabaWexLMBbXgBcf2CPn2qVrwzqf2zLnharyGvLjhzH5wyaGabaiaa+HcacqWG3bWDcqWGPbqAcqWG0baDcqWGObaAcqWGPbqAcqWGUbGBcqGGPaqkaaGccqGGOaakcqWGjbqsdaWgaaWcbaGaemiwaGfabeaakiabcYcaSiabdMeajnaaBaaaleaacqWGzbqwaeqaaOGaeiykaKIaeyypa0JaeGimaaJaeiOla4IaeGynauJaeG4naCdaaa@5543@, corMS/MS(within)(IX,IY)=0.61
 MathType@MTEF@5@5@+=feaafiart1ev1aaatCvAUfKttLearuWrP9MDH5MBPbIqV92AaeXatLxBI9gBaebbnrfifHhDYfgasaacH8akY=wiFfYdH8Gipec8Eeeu0xXdbba9frFj0=OqFfea0dXdd9vqai=hGuQ8kuc9pgc9s8qqaq=dirpe0xb9q8qiLsFr0=vr0=vr0dc8meaabaqaciGacaGaaeqabaqabeGadaaakeaaieaacqWFJbWycqWFVbWBcqWFYbGCdaqhaaWcbaGaemyta0Kaem4uamLaei4la8Iaemyta0Kaem4uamfabaWexLMBbXgBcf2CPn2qVrwzqf2zLnharyGvLjhzH5wyaGabaiaa+HcacqWG3bWDcqWGPbqAcqWG0baDcqWGObaAcqWGPbqAcqWGUbGBcqGGPaqkaaGccqGGOaakcqWGjbqsdaWgaaWcbaGaemiwaGLaeiilaWcabeaakiabdMeajnaaBaaaleaacqWGzbqwaeqaaOGaeiykaKIaeyypa0JaeGimaaJaeiOla4IaeGOnayJaeGymaedaaa@5762@) (Figure 8B) than those of peaks matching *between *clusters (corPMF(between)(IX,IY)=0.17
 MathType@MTEF@5@5@+=feaafiart1ev1aaatCvAUfKttLearuWrP9MDH5MBPbIqV92AaeXatLxBI9gBaebbnrfifHhDYfgasaacH8akY=wiFfYdH8Gipec8Eeeu0xXdbba9frFj0=OqFfea0dXdd9vqai=hGuQ8kuc9pgc9s8qqaq=dirpe0xb9q8qiLsFr0=vr0=vr0dc8meaabaqaciGacaGaaeqabaqabeGadaaakeaacqqGJbWycqqGVbWBcqqGYbGCdaqhaaWcbaGaemiuaaLaemyta0KaemOrayeabaGaeiikaGIaemOyaiMaemyzauMaemiDaqNaem4DaCNaemyzauMaemyzauMaemOBa4MaeiykaKcaaOGaeiikaGIaemysaK0aaSbaaSqaaiabdIfaybqabaGccqGGSaalcqWGjbqsdaWgaaWcbaGaemywaKfabeaakiabcMcaPiabg2da9iabicdaWiabc6caUiabigdaXiabiEda3aaa@4C14@, corMS/MS(between)(IX,IY)=0.04
 MathType@MTEF@5@5@+=feaafiart1ev1aaatCvAUfKttLearuWrP9MDH5MBPbIqV92AaeXatLxBI9gBaebbnrfifHhDYfgasaacH8akY=wiFfYdH8Gipec8Eeeu0xXdbba9frFj0=OqFfea0dXdd9vqai=hGuQ8kuc9pgc9s8qqaq=dirpe0xb9q8qiLsFr0=vr0=vr0dc8meaabaqaciGacaGaaeqabaqabeGadaaakeaacqqGJbWycqqGVbWBcqqGYbGCdaqhaaWcbaGaemyta0Kaem4uamLaei4la8Iaemyta0Kaem4uamfabaGaeeikaGIaemOyaiMaemyzauMaemiDaqNaem4DaCNaemyzauMaemyzauMaemOBa4MaeiykaKcaaOGaeiikaGIaemysaK0aaSbaaSqaaiabdIfaybqabaGccqGGSaalcqWGjbqsdaWgaaWcbaGaemywaKfabeaakiabcMcaPiabg2da9iabicdaWiabc6caUiabicdaWiabisda0aaa@4E34@) (Figure 8C). The correlation was determined for log transformed and root mean square scaled peak intensities. This indicated that the intensity of peaks could be employed for better discrimination of *within *and *between *cluster peak-list pairs. We have studied the performance of several intensity based measures: the covariance (cf. Methods Equation 10), the dot-product (cf. Methods Equation 9), the Manhattan and Euclidean distances (cf. Methods Equation 11), the relative distances Canberra and similarity index (cf. Methods Equation 12), and the sum of agreeing intensities (cf. Methods Equation 13).

### Peak intensity transformation

If two peaks match *within *a cluster, the peak intensities are very likely (except random matches) to be estimates of a number of the ions of the same peptide (PMF) or peptide fragment (MS/MS). These estimates might contain errors resulting from random noise, different levels of peptide fragmentation due to variations in *collision energy *and different signal-to-noise ratios due to varying concentrations of sample present [22].

The observed error can depend on the observed intensity. Thus, any statistical model would either directly account for the variances or transform the data so that the variances are approximately equal for all peak intensity levels. To be able to determine the best variance stabilising transformation, one can examine the *proportionate reduction in variation R*^2 ^[42], obtained by analysis of the model |Δ*I*| ~ *Ī *+ *Ī*^2^, where Δ*I *= *I*_*X *_- *I*_*Y *_are the residues and *Ī *= (*I*_*X *_+ *Y*_*Y*_)/2 represent the average peak intensity of two matching peaks. This model accounts for a correlation of variance and intensity (|Δ*I*| ~ *Ī*), unlike the naive model Δ*I *= *E*(Δ*I*) [43]. If the variance is stable, the naive model suffices, and the proportionate reduction in variation obtained with the complex model |Δ*I*| ~ *Ī *+ *Ī*^2 ^should be close to zero.

The Altman-Bland plots [43] in Figures 8B and 8C, show the residues (Δ*I *= *I*_*X *_- *I*_*Y*_) as a function of the average peak intensity *Ī *= (*I*_*X *_+ *I*_*Y*_)/2, where *I*_*X *_and *I*_*Y *_are the intensities of a matching peak pair (*X*, *Y*). The peak intensities are log-transformed and root mean square scaled (cf. Methods – Equation 22). Table 7 shows the adjusted *R*^2 ^of the model |Δ*I*| ~ *Ī *+ *Ī*^2 ^for various peak intensity transformations. The log-transformation gives the best variance stabilisation.

**Table 7:** The adjusted *R*^2 ^of the model |Δ*I*| ~ *Ī *+ *Ī*^2 ^for the raw, squared (Tabb et al. [22]) and log transformated peak intensities. PMF – PMF-data; MS/MS – MS/MS data.

	raw	sqrt	log
PMF	0.47	0.32	0.04
MS/MS	0.40	0.16	0.02

To elucidate to which extent the transformation influences the PAUC score, as compared to other factors, we kept the different transformations as factor levels of the pairwise peak-list comparison process. This wasdone despite the fact that the best transformation was determined by the analysis of the proportionate reduction of variance. In addition to the raw, root-squared and log-transformed intensities we included the ranking of the intensities [27] among the transformations studied by ANOVA.

## Abbreviations

• ANOVA – analysis of variance

• ROC – receiver operating characteristic curve.

• PAUC – partial area under the curve.

• TP – true positive.

• FP – false positive.

• FN – false negative.

• TN – true negative.

## Authors' contributions

HL, JG, KR, ML and RH gave initial input to the research.

WEW implemented the dissimilarities, evaluation framework, and designed the figures.

WEW, RH and PM planned and carried out the analysis.

WEW, ML, PM, KR, PG and JG wrote the manuscript.

AS provided the MS/MS dataset.

JG and PG provided the PMF datasets.

All authors contributed to the final version of the manuscript and approved it.

## Supplementary Material

Additional File 1 Column name and factor level definition of the following four tables. Click here for file

Additional File 2 The file contains the sensitivity and specificity PAUC obtained for peak-list comparison approaches based on binary measure and the PMF dataset. Click here for file

Additional File 3 The file contains the sensitivity and specificity PAUC obtained for peak-list comparison approaches based on measures considering peak intensities and the PMF dataset. Click here for file

Additional File 4 The file contains the sensitivity and specificity PAUC obtained for peak-list comparison approaches based on the binary measure based and the MS/MS dataset. Click here for file

Additional File 5 The file contains the sensitivity and specificity PAUC obtained for peak-list comparison approaches based on measures considering peak intensities and the MS/MS dataset. Click here for file
